# SFL-3D-cultured adipose mesenchymal cell-derived extracellular vesicles promote diabetic wound healing by alleviating microvascular endothelial senescence *via* PI3K/AKT/mTOR/4EBP1-mediated cap-dependent translation

**DOI:** 10.1093/burnst/tkag042

**Published:** 2026-06-22

**Authors:** Yunwei Wang, Zhihan Hu, Ao Shi, Peng Cao, Luyang Zhao, Xiaoyu Di, Yuchen Kang, Jiatong Wang, Li Gong, Wenjiao Chen, Gang Wang, Guangtong Cao, Liang Luo, Ruomei Zhao, Xi Zhang, Yi Liu

**Affiliations:** Department of Burn Plastic and Wound Repair Surgery, Lanzhou University Second Hospital, No. 82 Cuiyingmen, Lanzhou, Gansu 730030, China; Department of Plastic and Reconstructive Surgery, Xijing Hospital, Fourth Military Medical University, 127 West Changle Road, Xi’an, Shaanxi 710032, China; Key Laboratory of Stem Cells and Gene Drugs, Gansu Province, No. 333, Nanbinhe Road, Qilihe District, Lanzhou, Gansu 730030, China; Department of Burn Plastic and Wound Repair Surgery, Lanzhou University Second Hospital, No. 82 Cuiyingmen, Lanzhou, Gansu 730030, China; Key Laboratory of Stem Cells and Gene Drugs, Gansu Province, No. 333, Nanbinhe Road, Qilihe District, Lanzhou, Gansu 730030, China; Department of Burn Plastic and Wound Repair Surgery, Lanzhou University Second Hospital, No. 82 Cuiyingmen, Lanzhou, Gansu 730030, China; Key Laboratory of Stem Cells and Gene Drugs, Gansu Province, No. 333, Nanbinhe Road, Qilihe District, Lanzhou, Gansu 730030, China; Burns and Trauma Treatment Center, Affiliated Hospital of Jiangnan University, No. 1000 Hefeng Road, Binhu District, Wuxi, Jiangsu 214122, China; Department of Plastic and Reconstructive Surgery, Xijing Hospital, Fourth Military Medical University, 127 West Changle Road, Xi’an, Shaanxi 710032, China; Department of Burn Plastic and Wound Repair Surgery, Lanzhou University Second Hospital, No. 82 Cuiyingmen, Lanzhou, Gansu 730030, China; Department of Burn Plastic and Wound Repair Surgery, Lanzhou University Second Hospital, No. 82 Cuiyingmen, Lanzhou, Gansu 730030, China; Department of Burn Plastic and Wound Repair Surgery, Lanzhou University Second Hospital, No. 82 Cuiyingmen, Lanzhou, Gansu 730030, China; Department of Burn Plastic and Wound Repair Surgery, Lanzhou University Second Hospital, No. 82 Cuiyingmen, Lanzhou, Gansu 730030, China; Department of Burn Plastic and Wound Repair Surgery, Lanzhou University Second Hospital, No. 82 Cuiyingmen, Lanzhou, Gansu 730030, China; Department of Burn Plastic and Wound Repair Surgery, Lanzhou University Second Hospital, No. 82 Cuiyingmen, Lanzhou, Gansu 730030, China; Department of Burn Plastic and Wound Repair Surgery, Lanzhou University Second Hospital, No. 82 Cuiyingmen, Lanzhou, Gansu 730030, China; Department of Plastic and Reconstructive Surgery, Xijing Hospital, Fourth Military Medical University, 127 West Changle Road, Xi’an, Shaanxi 710032, China; Department of Burn Plastic and Wound Repair Surgery, Lanzhou University Second Hospital, No. 82 Cuiyingmen, Lanzhou, Gansu 730030, China; Key Laboratory of Stem Cells and Gene Drugs, Gansu Province, No. 333, Nanbinhe Road, Qilihe District, Lanzhou, Gansu 730030, China; Department of Plastic and Reconstructive Surgery, Xijing Hospital, Fourth Military Medical University, 127 West Changle Road, Xi’an, Shaanxi 710032, China; Department of Burn Plastic and Wound Repair Surgery, Lanzhou University Second Hospital, No. 82 Cuiyingmen, Lanzhou, Gansu 730030, China

**Keywords:** Diabetic foot ulcer, Extracellular vesicle, Adipose-derived mesenchymal stem cell, 3D culture, Microvascular endothelial senescence, PI3K/AKT/mTOR signaling, Cap-dependent translation, Wound healing, Diabetic wound

## Abstract

**Background:**

Diabetic foot ulcers (DFUs) are severe complications of diabetes, and treatment options for DFUs are limited. Current research on impaired angiogenesis in DFUs predominantly relies on generic endothelial cells, which inadequately reflect the pathophysiological microenvironment of the diabetic microvasculature. In contrast, this study focused specifically on human dermal microvascular endothelial cell (HDMEC) senescence as a central mechanism in DFU progression. We used a self-feeder layer 3D (SFL-3D) culture system to reprogram adipose-derived mesenchymal stem cells (ADSCs) to prepare functionally enhanced three-dimensional adipose stem cells (tdASCs) and their extracellular vesicles (tdASC-EVs) to mitigate HDMEC senescence and improve diabetic healing.

**Methods:**

EVs were isolated from SFL-3D-induced tdASCs and characterized. *In vitro*, high-glucose-induced HDMECs were treated with tdASC-EVs, after which senescence, oxidative stress, mitochondrial function, and angiogenesis were assessed. Pathway-specific inhibitors and a phosphorylation-mimetic 4EBP1 plasmid were used to validate the mechanistic involvement of the phosphatidylinositol 3-kinase/Ak strain transforming/mechanistic target of rapamycin/4E-binding protein 1 (PI3K/AKT/mTOR/4EBP1) axis and cap-dependent translation *via* m^7^GTP pull-down. *In vivo*, a fat graft model was used to assess the stem cell function of tdASCs, whereas a diabetic wound model was used to evaluate the therapeutic efficacy of tdASC-EVs.

**Results:**

tdASC-EVs significantly attenuated high-glucose-induced HDMEC senescence, oxidative stress, and mitochondrial dysfunction while increasing tube formation. Mechanistically, tdASC-EVs activated PI3K/AKT/mTOR signaling, increased 4EBP1 phosphorylation, and promoted eIF4E–eIF4G assembly. In diabetic mice, tdASC-EVs accelerated wound closure and increased microvascular density, which correlated with reduced p16 expression and increased CD31 and p-4EBP1 expression. In the Bama miniature pig diabetic large animal model, tdASC-EVs exhibited markedly superior therapeutic effects compared with those induced by conventional ADSC-EVs, as evidenced by significantly faster wound closure, greater neovascularization density, narrower scar width, and more orderly collagen deposition.

**Conclusions:**

tdASC-EVs ameliorated diabetic wound healing by targeting HDMEC senescence through PI3K/AKT/mTOR/4EBP1-mediated cap-dependent translational activation. The preclinical large animal data further confirmed the superior efficacy of tdASC-EVs over ADSC-EVs, highlighting the therapeutic potential of SFL-3D-modified EVs for DFU treatment.

## Graphical Abstract

**Graphical Abstract:**
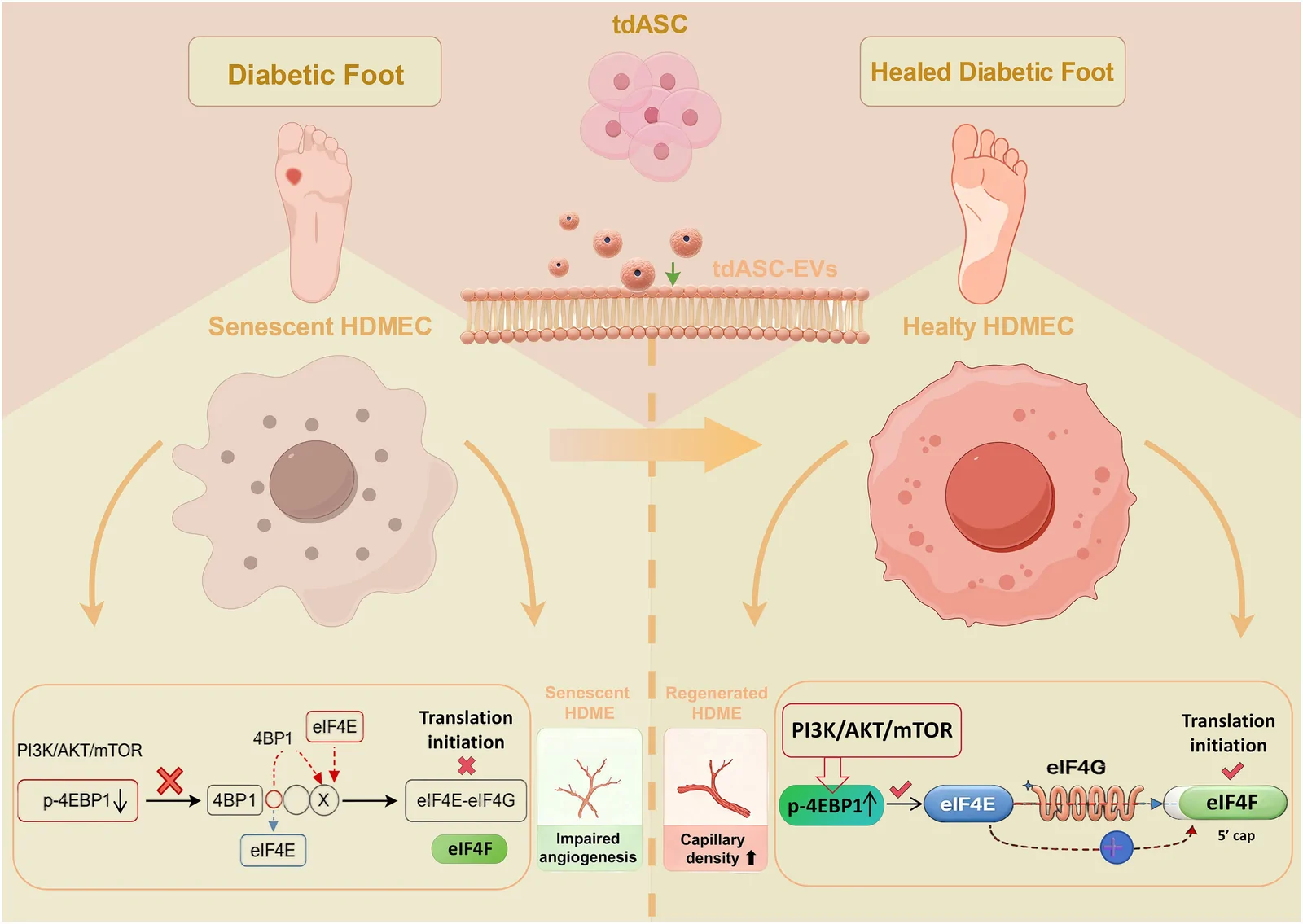

## Highlights

- Self-feeder layer 3D (SFL-3D) culture enhances adipose-derived mesenchymal stem cells (ADSC) stemness and extracellular vesicles (EV) yield, enriching tdASC-EVs with proregenerative protein cargo.
- Three-dimensional adipose stem cells (tdASC-EVs) activate the PI3K/AKT/mTOR/4EBP1 axis to restore p-4EBP1 levels and promote cap-dependent translation, thereby reversing high-glucose-induced microvascular endothelial senescence.
- In a diabetic porcine model, tdASC-EVs accelerate wound closure, increase neovascularization, and reduce scar formation more effectively than conventional ADSC-EVs.

## Background

Diabetic foot ulcers (DFUs) are among the most severe and debilitating complications of diabetes mellitus, with a globally increasing incidence and profound clinical and economic impacts [1, 2]. Current treatment strategies, including debridement, infection control, and advanced wound dressings, often yield unsatisfactory outcomes, leading to high rates of recurrence and amputation [3, 4]. The impaired healing process in DFUs is multifactorial and involves persistent inflammation, microvascular dysfunction, and cellular senescence. Accumulating evidence indicates that the senescence of vascular endothelial cells (ECs) plays a pivotal role in compromising angiogenesis and tissue repair in diabetic wounds [5–7].

Through single-cell ribonucleic acid (RNA) sequencing analysis of human DFU tissues, we observed significant alterations in microvascular EC subpopulations, accompanied by a pronounced senescent phenotype. Pathway enrichment further revealed the involvement of the phosphatidylinositol 3-kinase/Ak strain transforming/mechanistic target of rapamycin/4E-binding protein 1 (PI3K/AKT/mTOR/4EBP1) axis—a critical signaling cascade that regulates cell survival, proliferation, and protein translation—in this pathological process. These findings suggest that targeting endothelial senescence *via* this pathway may be a promising therapeutic strategy for DFUs.

Although extracellular vesicles (EVs) derived from adipose-derived mesenchymal stem cells (ADSCs) have shown considerable potential for enhancing diabetic wound healing through their proangiogenic and antisenescence effects, the therapeutic efficacy of conventional ADSC-EVs remains limited because of the gradual loss of stemness and reduced secretory function of ADSCs during *in vitro* expansion [8]. Previous studies have demonstrated that self-feeder layer 3D (SFL-3D) culture systems can significantly increase the biological activities and regenerative functions of stem cells, including dermal papilla cells (DPCs) and ADSCs [9–11]. The ADSC spheres obtained from SFL-3D-cultured three-dimensional adipose stem cells (tdASCs) were designated tdASCs. However, whether EVs derived from ADSCs expanded under SFL-3D conditions (tdASC-EVs) exhibit superior therapeutic effects—and through what mechanisms—remains entirely unexplored.

Therefore, this study aimed to investigate the therapeutic potential and underlying mechanisms of tdASC-EVs in diabetic wound healing. We hypothesized that tdASC-EVs can alleviate high-glucose-induced endothelial senescence and dysfunction by activating the PI3K/AKT/mTOR/4EBP1 signaling axis, thereby increasing cap-dependent translation initiation and promoting functional angiogenesis. Using both *in vitro* and *in vivo* models, we demonstrated that tdASC-EVs significantly mitigate cellular senescence, reduce oxidative stress, restore mitochondrial function, and improve the angiogenic capacity of human dermal microvascular endothelial cell (HDMECs). Furthermore, we validated the efficacy of tdASC-EVs in increasing fat graft survival and accelerating wound closure in diabetic mice. These findings provide novel insights into the application of SFL-3D culture technology for the production of highly functional EVs and highlight a previously unrecognized mechanism involving translational regulation in DFU treatment.

## Methods

### Patients and ethical approval

DFU periulcer skin tissue and normal foot (NF) skin tissue were collected from DFU patients and healthy controls, respectively. HDMECs were isolated from normal human skin tissue as previously described [12]. All human tissue collections were approved by the Ethics Committee of Lanzhou University Second Hospital (Approval No.: 2025A-558). Written informed consent was obtained from all participants.

### SFL-3D culture and tdASCs identification

ADSCs were obtained from the laboratory cell bank and maintained in α-MEM supplemented with 10% FBS for passages 1–5 under 5% CO₂ at 37°C. After five passages, the cells were switched to DMEM/F-12 supplemented with 10% FBS. ADSCs were dissociated for 5 min with StemPro™ Accutase™ Reagent (Gibco) to obtain a single-cell suspension. Afterwards, 5 × 10^4^ cells/cm^2^ were seeded in SFL-3D culture medium supplemented with 2% FBS, 5 ng/ml human bFGF, 2 ng/ml human EGF, 5 ng/ml human PDGF, 2 μg/ml heparin, 50 μg/ml L-ascorbic acid 2-phosphate sesquimagnesium salt hydrate, 100 U/ml penicillin, and 100 μg/ml streptomycin [9]. ADSCs cultured under these 3D conditions are referred to as tdASCs.

tdASCs were identified by immunofluorescence staining for CD90 and OCT4 (images acquired using a confocal microscope). Multipotent differentiation potential was assessed using adipogenic and osteogenic induction kits (Cyagen), followed by Oil Red O and Alizarin Red staining, respectively. Flow cytometry was performed to analyse the expression of mesenchymal stem cell markers (CD29, CD44, CD90, and CD105) and hematopoietic markers (CD39 and CD45) using a BD FACSVerse flow cytometer.

### Evaluation of ADSCs and tdASCs

Adipogenic and osteogenic differentiation were evaluated by Oil Red O and Alizarin Red staining, respectively. Cell viability was measured using a cell counting kit-8 (CCK-8) assay (Beyotime, China). The population doubling time (PDT) was calculated on the basis of cell counts over multiple passages [11]. The expression of stemness genes [Nanog, Sox2, Klf4, and alkaline phosphatase (ALP)] was analysed by quantitative reverse transcription polymerase chain reaction (qRT–PCR).

### EVs isolation and characterization

EVs were isolated from the conditioned media of ADSCs and tdASCs by differential ultracentrifugation [13]. EV morphology was examined by transmission electron microscopy (TEM), and the size distribution was analysed using NanoFCM. EV markers (CD9, CD63, and TSG101) were confirmed by Western blotting.

### Animal models

Fat graft model: nude mice (BALB/c-nu, 6 weeks old) were subcutaneously transplanted with 0.3 ml of human fat granules mixed with 0.1 ml of phosphate-buffered saline (PBS), ADSCs (1 × 10^6^ cells), tdASCs (1 × 10^6^ cells), ADSC-EVs (100 μg), or tdASC-EVs (100 μg) (*n* = 8) [14, 15]. Graft retention was evaluated by weight and volume at 4 weeks post-transplantation.

Full-thickness skin defect model: wild-type (WT) and db/db diabetic mice (6 weeks old) were anaesthetized, and a full-thickness excisional wound with a diameter of 1 cm was created on the dorsal skin. Wounds were treated topically with PBS or tdASC-EVs (100 μg in 0.1 ml of PBS) (*n* = 8). Wound closure was monitored periodically.

Large animal chronic wound model: This study used Bama miniature pigs weighing 20–25 kg. A diabetic state was induced as previously described [16] through a combination of high-energy diet feeding and multiple intravenous injections of streptozotocin (total dose of 125 mg/kg). Following the confirmation of a stable diabetic state, chronic, full-thickness skin wounds were surgically created on each animal according to the established protocol [17]. Briefly, six circular full-thickness wounds (2 cm in diameter) were created on the depilated dorsal skin—three on each side of the midline. The wounds were then divided into three treatment groups. Starting immediately post-wounding, each group received daily periwound subcutaneous injections for a duration of 4 weeks as follows: the control group received 2 ml of PBS; the ADSC-EV group received 2 ml of PBS containing 400 μg of ADSC-EVs; and the tdASC-EV group received 2 ml of PBS containing 400 μg of tdASC-EVs. Wound healing progression was monitored throughout the 4-week treatment period. All animal experiments were approved by the Animal Committee of Lanzhou University Second Hospital (Approval No: 2025-741) and were performed in accordance with the ARRIVE guidelines.

### Single-cell sequencing

Single-cell RNA sequencing data were downloaded from the GEO database (GSE165816), which included NF (*n* = 10) and DFU (*n* = 5) samples [18]. Data processing, normalization, clustering, and annotation were performed using Seurat (v5.3.0) in R (v4.5.1). Differential expression and pathway enrichment analyses were conducted using clusterProfiler, AnnotationDbi, org.Hs.eg.db, ggplot2, EnhancedVolcano, and ComplexHeatmap.

### Cell culture

HDMECs were cultured in EGM-2 MV medium (Lonza, USA) under normal glucose (5.5 mM) or high-glucose (25 mM) conditions for 3 days to simulate diabetic hyperglycemia.

### Cell proliferation assay

Cell viability was assessed using a CCK-8 kit (Beyotime, China) according to the manufacturer’s instructions. PDT was calculated on the basis of cell counts over multiple passages.

### Senescence-associated β-galactosidase staining

Cellular senescence was evaluated using a Senescence β-Galactosidase Staining Kit (Beyotime, C0602) according to the manufacturer’s instructions. In brief, after the indicated treatments were applied, the cells were fixed and incubated with β-galactosidase staining solution at 37°C overnight in a CO₂-free environment. The following day, the cells were imaged under a light microscope. Senescent cells were identified by the presence of distinctive blue cytoplasmic staining.

### Immunofluorescence staining

Cells or tissue sections were fixed, permeabilized, and incubated with primary antibodies against OCT4 (CST, 2750; 1:200), CD90 (Proteintech, 66766-1-Ig; 1:200), CD31 (CST, 15585; 1:300), 4EBP1 (Proteintech, 60246-1-Ig; 1:200), p16 (CST, 23200; 1:200), and PPARγ (Proteintech, 16643-1-AP; 1:200), followed by fluorophore-conjugated secondary antibodies. The nuclei were counterstained with 4′6-diamidino-2-phenylindole (DAPI). Images were acquired using a confocal microscope. Quantitative analysis of immunofluorescence images was performed using ImageJ software.

### qRT–PCR

Total RNA was extracted using TRIzol reagent (Invitrogen). cDNA was synthesized using PrimeScript RT Master Mix (Takara). qRT–PCR was performed with SYBR Green (Roche) on a QuantStudio system. Gene expression was normalized to that of GAPDH. The primer sequences for the target genes are detailed in Supplementary Table S1.

### Mitochondrial membrane potential assay

The mitochondrial membrane potential was measured using a JC-1 assay kit (Beyotime, C2006). After treatment, the HDMECs were incubated with JC-1 working solution at 37°C for 20 min. The cells were subsequently washed and analysed by fluorescence microscopy or flow cytometry (BD FACSVerse). The red/green fluorescence ratio was calculated to assess membrane depolarization.

### Oxidative stress detection

8-OHdG immunofluorescence staining: HDMECs were fixed, permeabilized, and stained with an anti-8-OHdG antibody (Abcam, ab48508; 1:200) followed by incubation with a fluorophore-conjugated secondary antibody. The nuclei were counterstained with DAPI. Images were acquired by confocal microscopy and analysed with ImageJ.

8-OHdG ELISA: The cell culture supernatants were collected and centrifuged. 8-OHdG levels were quantified using a Human 8-OHdG ELISA Kit (Elabscience) according to the manufacturer’s instructions. The absorbance was measured at 450 nm.

### Protein extraction and western blotting

Proteins were extracted using RIPA buffer supplemented with protease and phosphatase inhibitors. The samples were separated by SDS–PAGE, transferred to PVDF membranes, and probed with antibodies against p-4EBP1 (Proteintech, 81 812–4-RR; 1:2000), 4EBP1 (CST, 39788; 1:2000), p-AKT (Proteintech, 66 444–1-Ig; 1:2000), AKT (Proteintech, 10 176–2-AP; 1:2000), p-mTOR (CST, 5536; 1:2000), mTOR (CST, 2792; 1:2000), p-PI3K (CST, 4228; 1:2000), and PI3K (CST, 4292; 1:2000). The blots were developed using ECL reagent.

### Plasmid transfection

The full-length and hyperphosphorylated mutant 4EBP1 (4EBP1-2xAsp) was cloned and inserted into the pcDNA3.1-3xFlag-C vector (Fenghui Bio). HDMECs were transfected with plasmids or siRNAs using Lipofectamine 3000 or RNAiMax (Invitrogen) according to the manufacturer’s instructions [19].

### 7-Methylguanosine 5′-triphosphate pull-down assay

Cap-dependent translation complex formation was analysed using m^7^GTP Agarose Beads (Jena Bioscience, AC-155S). To validate the specificity of the pull-down, two critical control assays were performed in parallel: (i) bead control: cell lysates were incubated with plain agarose beads (without conjugated m^7^GTP) to assess nonspecific binding to the bead matrix; and (ii) competition control: lysates were preincubated with an excess of free m^7^GDP (100 μM, Jena Bioscience, NU-401) for 30 min at 4°C prior to incubation with m^7^GTP beads to competitively disrupt the specific interaction between eIF4E and the immobilized cap analogue. HDMEC lysates were incubated with beads at 4°C for 2 h. The beads were subsequently washed, and the bound proteins were eluted for Western blot analysis of eIF4E (Proteintech, 11 149–1-AP; 1:2000), 4EBP1 (CST, 39788; 1:2000), and eIF4G (CST, 2498; 1:2000). Input lysates (10% of the total amount used for each pull-down reaction) served as loading controls and for normalization. The signal intensities of proteins in the pull-down samples were quantitatively normalized to their corresponding levels in the input samples from the same lysate to control for variations in total protein expression.

### Proteomic profiling of EVs

Comparative proteomic analysis of EVs derived from tdASCs and conventional ADSCs was performed by Guangzhou Genedenovo Biotechnology Co., Ltd. Equal amounts of protein from each sample were reduced, alkylated, and digested with trypsin using the filter-aided sample preparation method. Peptides were analysed by LC–MS/MS on a Q exactive HF instrument, and raw data were searched against the UniProt human database using MaxQuant with an FDR < 1%. Label-free quantification was used to identify differentially expressed proteins [DEPs; fold change (FC) >2, *P* < 0.05]. Hierarchical clustering, volcano plots, and GO/KEGG enrichment analyses were performed in R.

### Histopathological staining

The tissue sections were stained with hematoxylin and eosin (H&E) for general morphology and Masson’s trichrome for collagen deposition, following standard protocols.

### Statistical analysis

Normality was tested using the Shapiro–Wilk test. Normally distributed data are presented as the mean ± SD and were analysed by Student’s *t* test (two groups) or one-way or two-way analysis of variance with Tukey’s *post hoc* test (multiple groups). Nonnormally distributed data are presented as medians (IQRs) and were analysed by the Kruskal–Wallis test with Dunn’s post hoc test. Statistical significance was set at ^*^P < 0.05, ^**^P < 0.01, and ^***^P < 0.001. All analyses were performed using GraphPad Prism 9.0.

## Results

### Isolation and characterization of tdASCs cultured in a SFL-3D system

**Figure 1:**
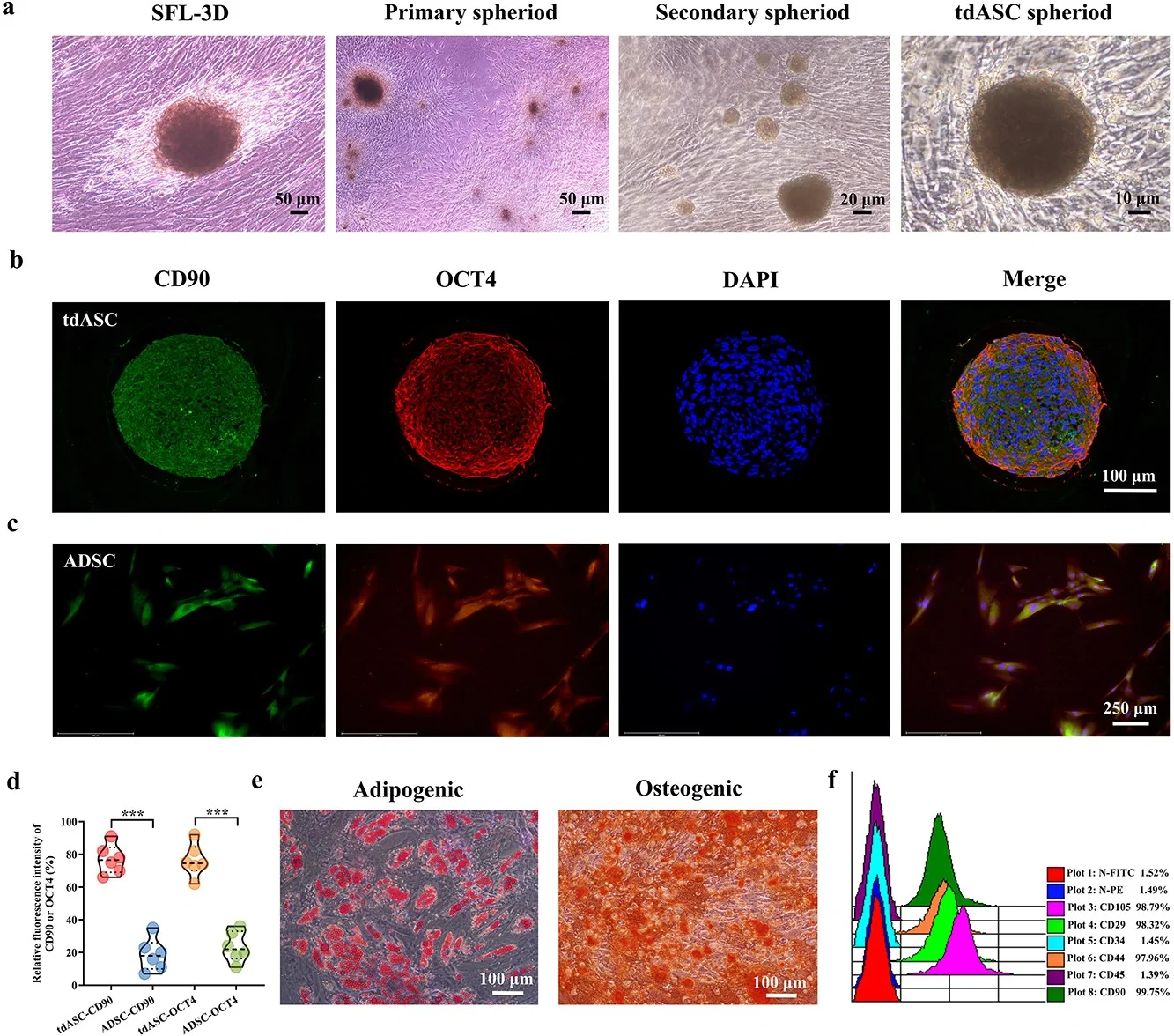
Culture and identification of tdASCs. (**a**) Formation process of tdASC spheres in the SFL-3D culture system. Scale bar: 10 μm, 20 μm, and 50 μm. (**b** and **c**) Laser confocal imaging of immunofluorescence staining results for adipose stem cell markers in tdASCs or ADSCs. Scale bar: 100 μm and 250 μm. (**d**) Violin plots showing the statistical analysis of relative fluorescence intensities for CD90 and OCT4 in tdASCs and ADSCs. (**e**) Adipogenic and osteogenic staining results of tdASCs. Scale bar: 100 μm. (**f**) Assessment of the expression of mesenchymal stem cell markers in tdASCs using flow cytometry. *SFL-3D* self-feeder layer 3D, *tdASCs* three-dimensional adipose stem cells, *ADSCs* adipose-derived mesenchymal stem cells

We first characterized the spheroid formation process of ADSCs cultured in the SFL-3D system. The ADSCs progressed through stages of primary and secondary spheroid formation, ultimately generating tdASC spheres that were semisuspended above the adherent ADSC monolayer (Figure 1a). We subsequently identified tdASCs by immunofluorescence staining for the relatively specific adipose stem cell markers CD90 and OCT4, which were highly expressed in these spheroids (Figure 1b). Parallel immunofluorescence staining for these markers was also performed in ADSCs (Figure 1c). Quantitative comparison revealed that the fluorescence intensities of both CD90 and OCT4 were significantly greater in tdASCs than in ADSCs (Figure 1d). Furthermore, tdASCs exhibited a multipotent differentiation capacity, as demonstrated by positive staining for both adipogenic and osteogenic lineages (Figure 1e). Additionally, flow cytometric analysis confirmed that tdASCs were positive for classic mesenchymal stem cell markers (CD29, CD44, CD90, and CD105) and negative for hematopoietic markers (CD39 and CD45), which was consistent with typical stem cell surface antigen profiles (Figure 1f).

### Enhanced stem cell properties and functions of tdASCs *in vitro*

**Figure 2:**
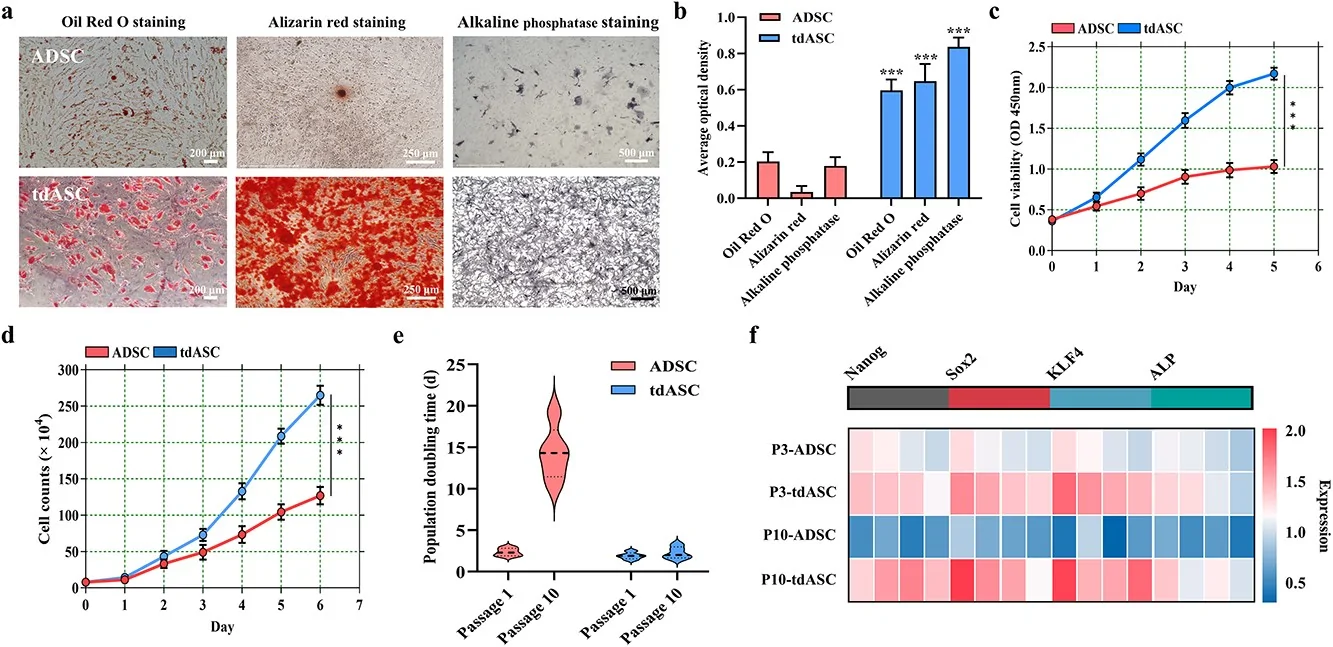
Characteristics of the directional differentiation ability, proliferation ability and stemness retention function of tdASCs *in vitro*. (**a**) Comparison of the Oil Red O staining, alizarin red staining and ALP staining results; scale bar: 200 μm, 250 μm, and 500 μm. (**b**) Statistical analysis of Oil Red O staining, alizarin red staining and ALP staining. (**c**) CCK-8 assay. The proliferation of the tdASC cell line significantly increased; *n* = 5; ^**^P < 0.01. (**d**) Plot showing the numbers of tdASCs and ADSCs; *n* = 5; ^**^P < 0.01. (**e**) Plot of the PDT of tdASC and ADSC self-renewing cultures; ns, not statistically significant; *n* = 5; ^***^P < 0.001. (f) qRT–PCR measurements of stem cell marker genes (Nanog, Sox2, Klf4 and ALP) in ADSCs and tdASCs. Ns, not statistically significant; *n* = 4; ^***^P < 0.001. *tdASCs* three-dimensional adipose stem cells, *ADSCs* adipose-derived mesenchymal stem cells, *ALP* alkaline phosphatase, *PDT* population doubling time

We systematically compared the stem cell functionalities of tdASCs and conventional ADSCs *in vitro*, focusing on directed differentiation potential, proliferative capacity, and stemness maintenance. Quantitative analysis of differentiation revealed that compared with passage-matched ADSCs, tdASCs were significantly more positive for Oil Red O, Alizarin Red, and ALP staining, indicating enhanced adipogenic, osteogenic, and early osteogenic differentiation capabilities, respectively (Figure 2a and b). Moreover, tdASCs demonstrated markedly increased cellular viability, as measured *via* a CCK-8 assay (Figure 2c), along with significantly increased proliferative capacity and reduced PDT, confirming their superior expansion potential (Figure 2d and e). To further evaluate stemness retention, we analysed the expression of key pluripotency genes—Nanog, Sox2, Klf4, and ALP—in both P3 and P10 cells. The heatmap of the qRT–PCR results clearly indicated that compared with ADSCs, tdASCs maintained significantly elevated expression levels of these stemness markers (Figure 2f). In addition, evaluation of EV biogenesis and secretion capacity revealed that compared with ADSCs, tdASCs possessed greater EV secretory ability and higher EV yield (Figure S1, see online supplementary material). Collectively, these results demonstrate that compared with conventional ADSCs, tdASCs exhibit substantially enhanced stem cell activity and functionality *in vitro*.

### Enhanced stemness and function of tdASCs in promoting adipogenesis *in vivo*

**Figure 3:**
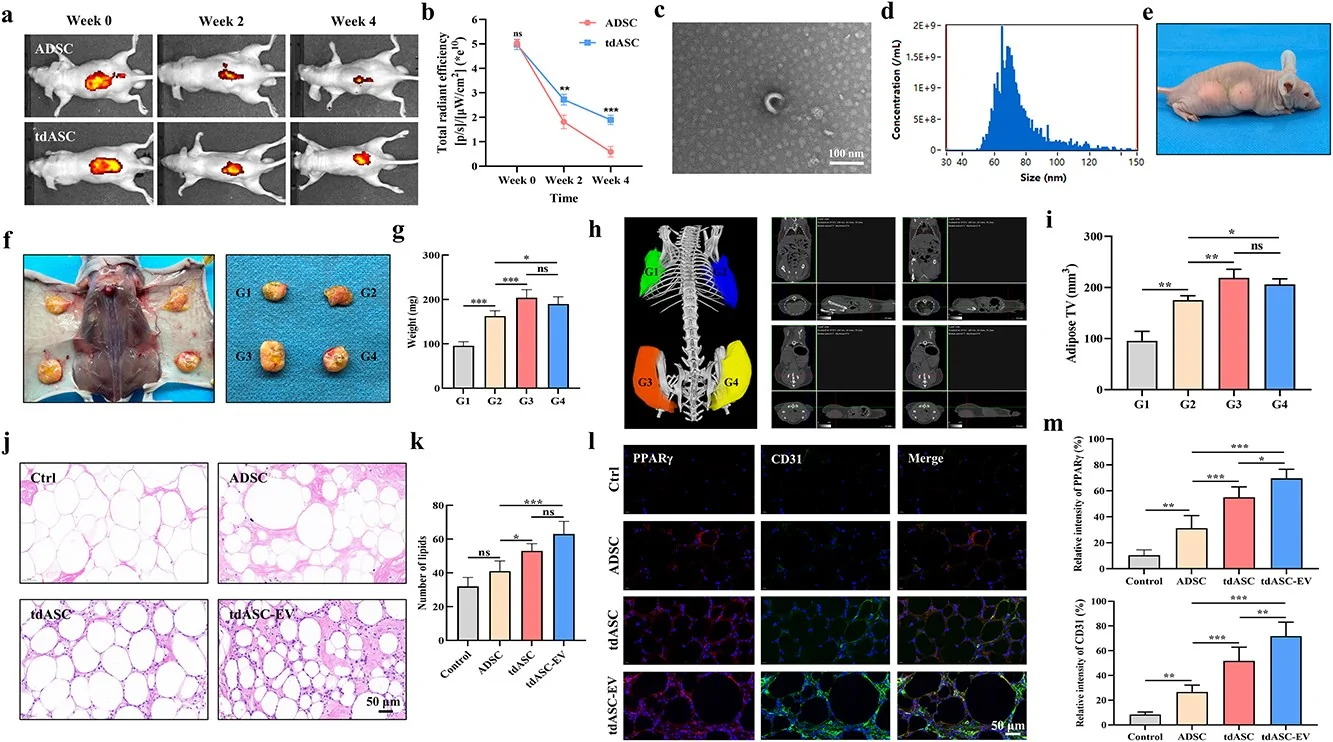
Identification of the ability of tdASCs and tdASC-EVs to promote lipogenesis and angiogenesis *in vivo*. (**a** and **b**) An animal imaging system was used to determine the survival of ADSCs and tdASCs after transplantation, and the results were statistically analysed. (**c**) Ultrastructures of tdASC-EVs were visualized *via* TEM. Scale bar: 100 nm. (**d**) The particle size distribution of tdASC-EVs was determined using NanoFCM. (**e** and **f**) Gross and anatomical images of the four groups of animals 4 weeks after the transplantation of granular fat into the nude mice. G1 represents the simple granular fat transplantation group, G2 represents the ADSC mixed granular fat transplantation group, G3 represents the tdASC mixed granular fat transplantation group, and G4 represents the tdASC-EV mixed granular fat transplantation group. (**g**) Statistical analysis of retention quality after fat transplantation in the four groups. (**h** and **i**) Statistical analysis of 3D CT images and retained volume after fat transplantation in the four groups. (**j** and **k**) H&E staining and statistical analysis of tissue sections from each group after fat transplantation. Scale bar: 50 μm. (**l** and **m**) Immunofluorescence staining and statistical analysis of lipogenic markers and angiogenesis markers in tissue sections from each group after fat transplantation. *n* = 8, ^*^P < 0.5, ^**^P < 0.01, and ^***^P < 0.001. G1: PBS; G2: ADSCs; G3: tdASCs; G4: tdASC-EVs. *tdASCs* three-dimensional adipose stem cells, *ADSCs* adipose-derived mesenchymal stem cells, *EV* extracellular vesicle, *TEM* transmission electron microscopy, *H&E* hematoxylin and eosin, *PBS* phosphate-buffered saline

To evaluate the *in vivo* vitality and function of tdASCs, we subcutaneously transplanted CM-DiL-labeled ADSCs and tdASCs into nude mice. Imaging after 4 weeks revealed that compared with that of conventional ADSCs, the survival rate of tdASCs was significantly greater (Figure 3a and b). To further investigate the functional mechanism, we isolated tdASC-derived EVs (tdASC-EVs), which exhibited a typical cup-shaped morphology in TEM and a homogeneous size distribution according to NanoFCM analysis (Figure 3c and d). We then conducted fat transplantation experiments using four treatment groups: granular fat mixed with PBS (G1), ADSCs (G2), tdASCs (G3), or tdASC-EVs (G4). After 4 weeks, graft retention was measured by weight and volume. Compared with the G1 control group, the G2 group had increased graft weight, whereas compared with the G2 group, the G3 and G4 groups exhibited significantly greater graft retention (Figure 3e–g). Consistently, micro-CT and 3D reconstruction confirmed that the graft volumes in the G3 and G4 groups were substantially greater than those in the G2 and G1 groups (Figure 3h and i). Histological analysis with H&E staining further demonstrated a greater number of intact lipid droplets in grafts from the G3 and G4 groups than in those from the G1 and G2 groups (Figure 3j and k). Immunofluorescence staining for the adipogenic marker PPARγ and the endothelial marker CD31 revealed stronger expression in G3 and G4, with the highest levels observed in the tdASC-EV-treated group (G4) (Figure 3l and m). Importantly, in a comparative study, tdASC-EVs outperformed ADSC-EVs in enhancing graft vascularization and survival (Figure S2, see online supplementary material). These results demonstrate that compared with ADSCs, tdASCs possess superior stem cell functionality *in vivo* and that tdASC-secreted EVs significantly promote fat graft survival by enhancing vascularization and adipogenic maintenance.

### Single-cell profiling reveals endothelial dysregulation in DFUs

**Figure 4:**
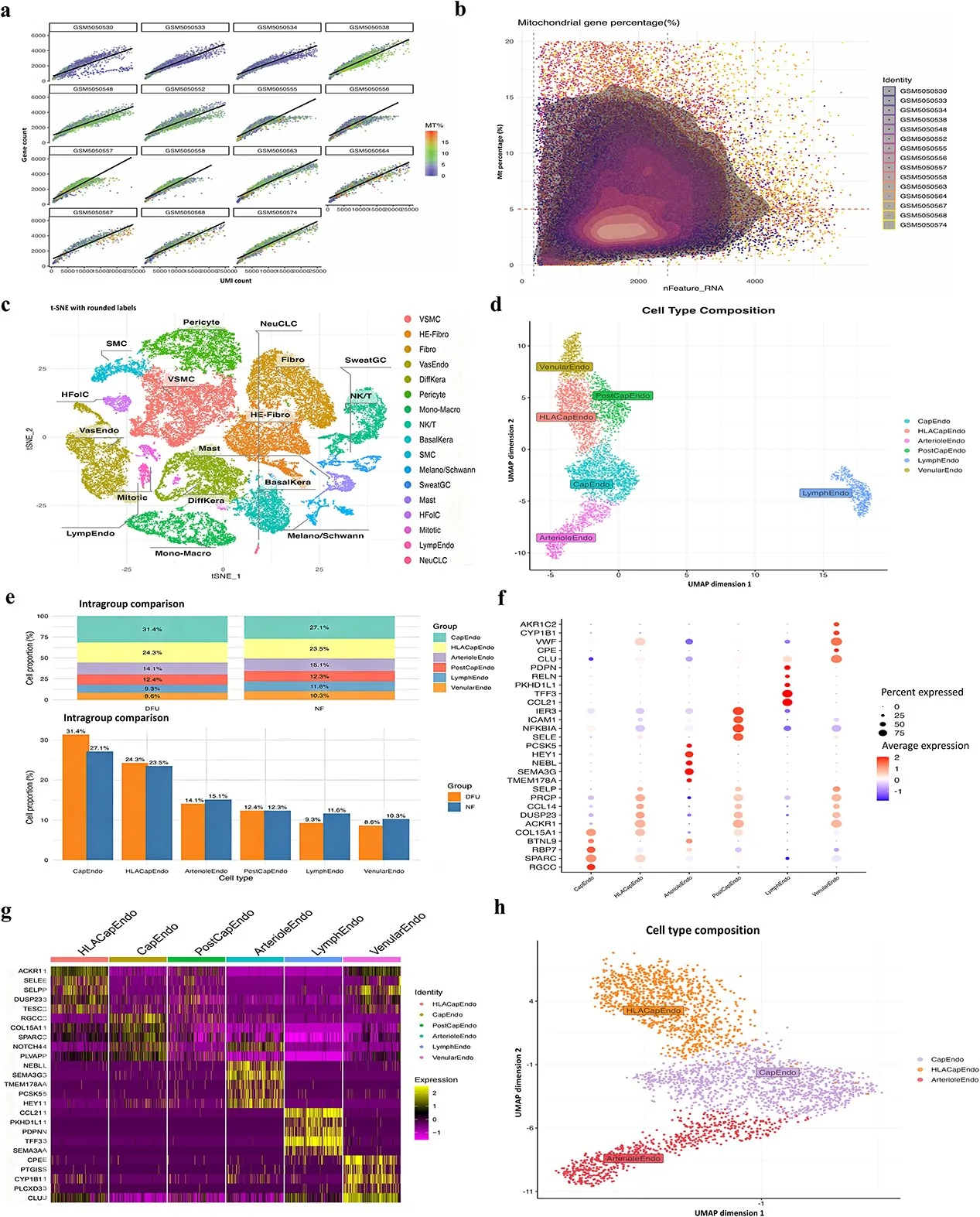
Single-cell atlas analysis of skin from NF and DFU patients. (**a**) Scatter plot showing the correlation between the number of genes (nFeature_RNA) and UMIs (nCount_RNA) per cell across the 12 selected samples (indicating a strong positive correlation). (**b**) Scatter plot showing the relationship between the number of detected genes (nFeature_RNA) and the percentage of mitochondrial genes (percent.mt) per cell. (**c**) t-SNE plot displaying 16 clusters identified by dimension reduction and clustering analysis and annotated using CellMarker2.0 and a literature review. (**d**) ECs (“VasEndo” and “LympEndo”) were extracted, reclustered, and annotated, which revealed 6 subtypes: ArterioleEndo, CapEndo, HLACapEndo, PostCapEndo, VenularEndo, and LymphEndo. (**e**) Comparison of endothelial subtype proportions between the DFU and NF groups revealed a significant reduction in the number of ArterioleEndo, VenularEndo, and LymphEndo cells in the DFU group, whereas the number of CapEndo cells increased. (**f**) Bubble plot displaying the top 5 marker genes for each endothelial subtype. (**g**) Heatmap showing genes that were differentially expressed across endothelial subtypes. (**h**) Focusing on microvascular dysfunction relevant to impaired DFU healing (neovascularization, microcirculation), subsequent analysis utilized the ArterioleEndo, CapEndo, and HLACapEndo subtypes, which were collectively designated MicrovascularEndo. *DFU* diabetic foot ulcers, *NF* normal foot, *ECs* endothelial cells

Having established the strong therapeutic potential of tdASC-EVs in enhancing vascularization and graft survival, we next investigated their mechanism of action in diabetic wound healing. We hypothesized that tdASC-EVs may ameliorate DFU impairment by modulating dysfunctional ECs. To systematically characterize the pathological cell landscape in DFU skin, we constructed a comprehensive single-cell atlas of human skin using samples from both normal feet and patients with DFUs (Figure 4). Initial quality control metrics confirmed robust data quality across the 12 integrated samples. Specifically, a strong positive correlation was observed between the number of genes detected per cell (nFeature_RNA) and the number of unique molecular identifiers (nCount_RNA) (Figure 4a). Furthermore, the relationship between nFeature_RNA and the percentage of mitochondrial transcripts (percent.mt) was visualized, which indicated minimal contamination from low-viability cells (Figure 4b). Unsupervised dimension reduction (t-SNE) and clustering of all cells revealed 16 distinct cellular clusters (Figure 4c). These clusters were meticulously annotated to major skin cell types using a combination of the CellMarker2.0 database and manual curation based on established marker genes reported in the literature.

To specifically investigate EC heterogeneity, we isolated cells annotated as vascular endothelial (VasEndo) and lymphatic endothelial (LympEndo) cells. This EC subset underwent independent reclustering, dimension reduction, and detailed reannotation. This high-resolution analysis revealed six distinct endothelial subtypes (Figure 4d): ArterioleEndo (arteriolar ECs), CapEndo (capillary ECs), HLACapEndo (HLA-high expressing capillary ECs), PostCapEndo (postcapillary venule ECs), VenularEndo (venular ECs), and LymphEndo (lymphatic ECs). Comparative analysis of endothelial subtype proportions revealed significant differences between the DFU and NF groups (Figure 4e). Notably, the proportions of ArterioleEndo, VenularEndo, and LymphEndo cells were significantly lower in DFU skin than in NF skin. Conversely, the proportion of CapEndo cells was increased in the DFU group. The distinct molecular identities of these endothelial subtypes were confirmed by visualizing the top five marker genes for each subtype using a bubble plot (Figure 4f). Further characterization through differential gene expression analysis highlighted unique transcriptional profiles across the subtypes, which were visualized in a heatmap (Figure 4g).

Given the critical clinical manifestations of impaired DFU healing, namely, a compromised blood supply, microcirculatory failure, and deficient neovascularization, which are mechanistically linked to disruptions in fundamental endothelial functions such as proliferation and migration, we refined our focus for subsequent mechanistic analyses. On the basis of this pathophysiological relevance to microvascular dysfunction, we excluded the LymphEndo and VenularEndo/PostCapEndo subtypes associated with the lymphatic and venous endothelium. We retained the ArterioleEndo, CapEndo, and HLACapEndo subtypes, which collectively represented the core microvascular endothelial compartment, and aggregated them under the designation MicrovascularEndo for downstream functional investigation (Figure 4h).

### tdASCs–EVs attenuate high-glucose-induced senescence and dysfunction in HDMECs

**Figure 5:**
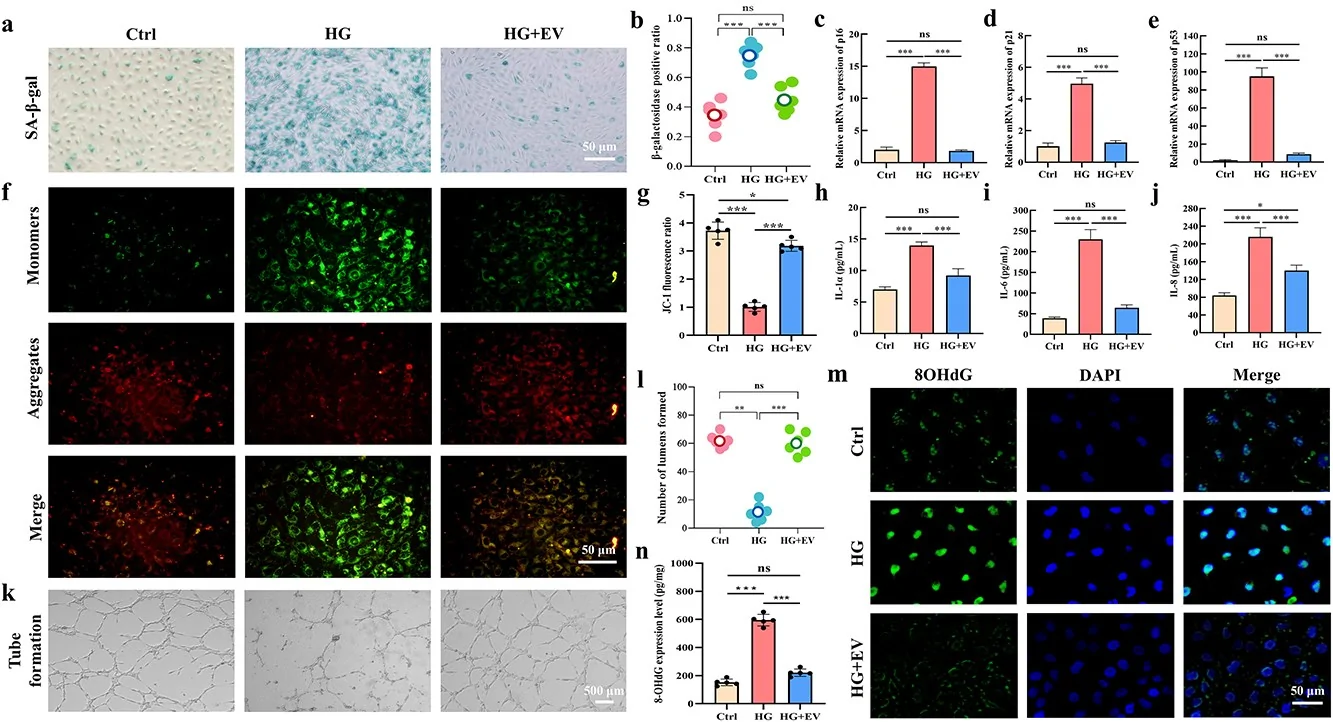
tdASC-EVs alleviate high-glucose-induced HDMEC senescence and oxidative stress damage. (**a** and **b**) β-galactosidase staining results and statistical analysis of HDMECs in each treatment group. Scale bar: 50 μm. (**c–e**) QRT–PCR was used to measure the expression of senescence markers in each treatment group. (**f–g**) Statistical analysis of JC-1 monomer fluorescence staining and JC-1 polymer fluorescence staining results and the fluorescence intensity of the HDMECs in each treatment group. Scale bar: 50 μm. (**h–j**) The expression of SASP components in each treatment group was assessed by ELISA. (**k–l**) Experimental results and statistical analysis of the lumen formation of HDMECs in each treatment group. Scale bar: 50 μm. (**m**) Staining intensity of 8-OHdG in HDMECs in each treatment group. Scale bar: 50 μm. (**n**) Levels of 8-OHdG in the culture supernatants of HDMECs in each treatment group. *n* = 6; ^*^P < 0.5, ^**^P < 0.01, and ^***^P < 0.001. *SASP* senescence-associated secretory phenotype, *tdASC* three-dimensional adipose stem cell, *EV* extracellular vesicle, *Ctrl* control, *HG* high glucose, *HDMECs* human dermal microvascular endothelial cells

To investigate the cytoprotective effects of tdASC-EVs, we first established an *in vitro* model of diabetic stress by culturing HDMECs under high-glucose conditions. Subsequent treatment with tdASC-EVs significantly mitigated high-glucose-induced cellular senescence, as evidenced by a reduction in SA-β-galactosidase activity to levels comparable to those of the normal control (Figure 5a and b). In parallel, high-glucose stimulation markedly upregulated the mRNA expression of key senescence markers (p16, p21, and p53), and this upregulation was effectively suppressed by tdASC-EV administration (Figure 5c–e). Assessment of mitochondrial membrane potential using JC-1 staining further revealed that tdASC-EVs notably reversed high-glucose-induced mitochondrial dysfunction, confirming an improvement in cellular bioenergetics (Figure 5f and g). Moreover, the elevated secretion of SASP factors triggered by high glucose was also significantly reduced by tdASC-EV treatment (Figure 5h–j). Functional recovery was further evidenced in tube formation assays, where tdASC-EVs restored the impaired angiogenic capacity of HDMECs under high-glucose conditions (Figure 5k and l). Finally, tdASC-EVs significantly alleviated oxidative stress, as shown by decreased intracellular 8-OHdG staining and reduced 8-OHdG levels in the culture supernatant (Figure 5m and n).

### Enrichment analysis of senescence-associated pathways and hub genes in the DFU microvascular endothelium

**Figure 6:**
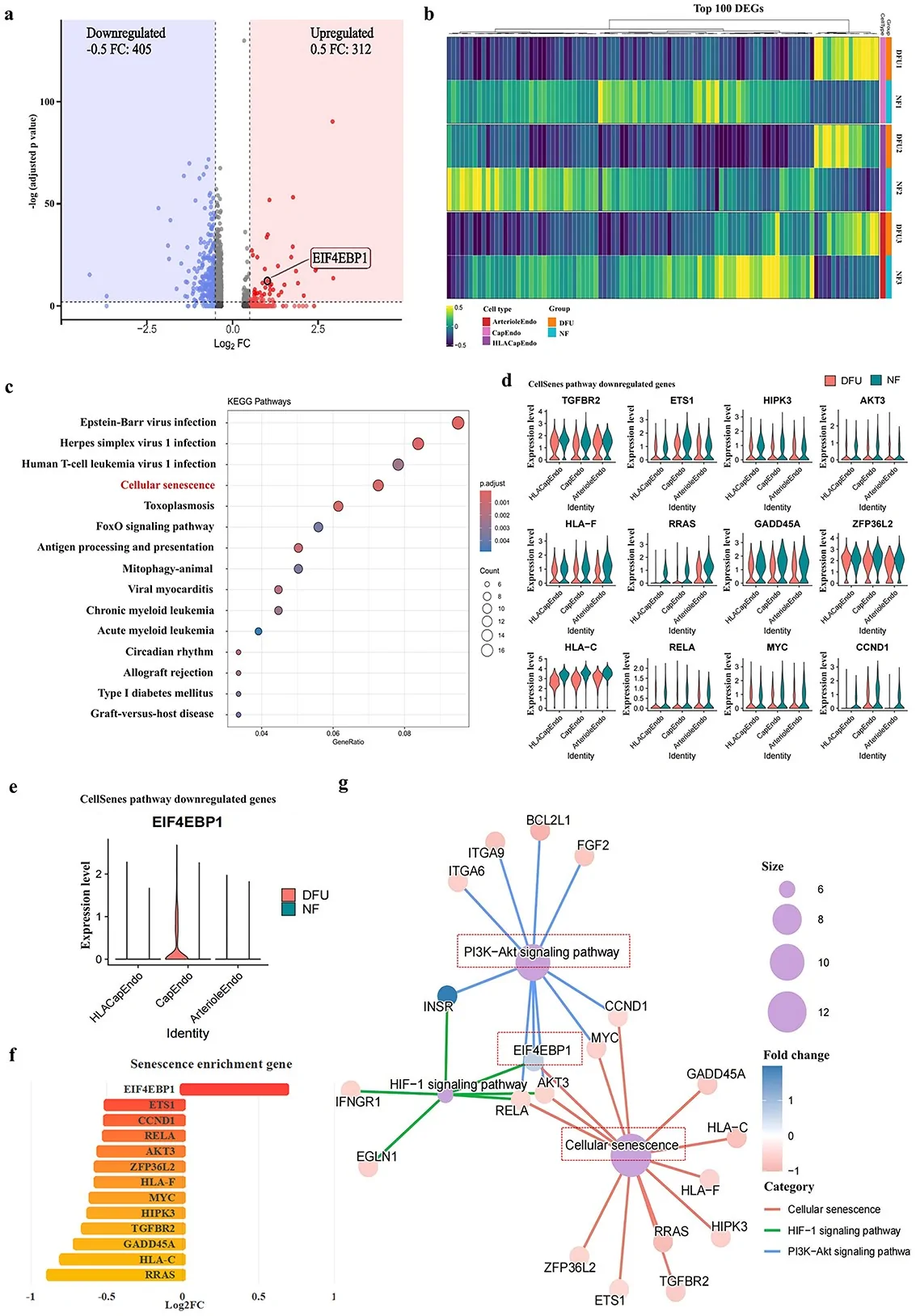
Enrichment analysis identifies senescence-associated pathways and hub genes in the DFU microvascular endothelium. (**a**) Volcano plot depicting DEGs in MicrovascularEndo cells between the DFU and NF groups reveals 573 upregulated genes and 887 downregulated genes. (**b**) Heatmap showing DEGs in the DFU versus NF groups for the ArterioleEndo, CapEndo, and HLACapEndo subtypes. (**c**) KEGG enrichment analysis revealed immune microenvironment dysregulation in ECs, including impaired antigen presentation, chronic inflammation, impaired cell adhesion/migration, and cellular senescence. (**d**) Violin plots showing differences in the expression of genes enriched in the cellular senescence pathway (KEGG:04218) between the DFU and NF groups across the ArterioleEndo, CapEndo, and HLACapEndo subtypes. (**e**) Violin plot indicating specific upregulation of EIF4EBP1 (4EBP1) in CapEndo cells from DFU patients. (**f**) Ranked list of senescence-associated significant DEGs in HDMECs. (**g**) Pathway network enrichment diagram demonstrating the central role of EIF4EBP1 in cellular senescence, PI3K–Akt signaling, and HIF-1 signaling. *HG* high glucose, *HDMECs* human dermal microvascular endothelial cells, *DFU* diabetic foot ulcers, *NF* normal foot, *ECs* endothelial cells, *AKT* Ak strain transforming, *FC* fold change, *DEGs* differentially expressed genes

Transcriptomic profiling of the aggregated MicrovascularEndo compartment revealed substantial gene expression alterations in DFU skin compared with those in NF skin. Specifically, differential expression analysis revealed a total of 1460 significantly dysregulated genes (DEGs), including 573 upregulated genes and 887 downregulated genes, in DFU samples, as shown in a volcano plot (Figure 6a). To investigate subtype-specific contributions to this dysregulation, we next analysed DEGs within each microvascular endothelial subtype (ArterioleEndo, CapEndo, and HLACapEndo). A heatmap clearly revealed distinct patterns of gene dysregulation across these three subtypes (Figure 6b).

Pathway enrichment analysis of the MicrovascularEndo DEGs provided critical insights into the underlying biological dysfunction. KEGG analysis revealed robust immune microenvironment dysregulation in DFU ECs, characterized by enrichment of pathways associated with impaired antigen processing and presentation (hsa04612) and chronic inflammation (hsa05169). Furthermore, pathways linked to dysfunctional cell adhesion and migration (hsa04810) were significantly enriched. Most notably, the results of the analysis revealed highly significant enrichment of the cellular senescence pathway (hsa04218), suggesting that ECs in diabetic wounds acquire a senescent phenotype (Figure 6c). To validate this senescence signature at the cellular level, we examined the expression of core genes involved in the cellular senescence pathway (hsa04218). Violin plots revealed significant differences in the expression of these senescence-associated genes between the DFU and NF groups across all three microvascular endothelial subtypes (Figure 6d). Strikingly, violin plot analysis revealed a specific and significant upregulation of eukaryotic translation initiation factor 4E-binding protein 1 (EIF4EBP1) expression exclusively within the CapEndo subtype of DFU patients (Figure 6e). Focusing specifically on clinically relevant HDMECs, we generated a ranked list of the most significant senescence-associated DEGs identified in the DFU versus NF comparison (Figure 6f). Among these top-ranked genes, the translational regulator EIF4EBP1 (4EBP1) exhibited particularly compelling expression dynamics. Given the prominence of 4EBP1 in our senescence signature and its CapEndo-specific induction, we investigated its broader functional role using pathway interaction network analysis. This analysis identified 4EBP1 as a pivotal regulator, demonstrating its central involvement not only in the cellular senescence pathway (hsa04218) but also in the PI3K-Akt signaling pathway (hsa04151) and the HIF-1 signaling pathway (hsa04066) (Figure 6g).

### Experimental validation of p-4EBP1/4EBP1 dysregulation in DFUs and high-glucose-induced HDMECs

**Figure 7:**
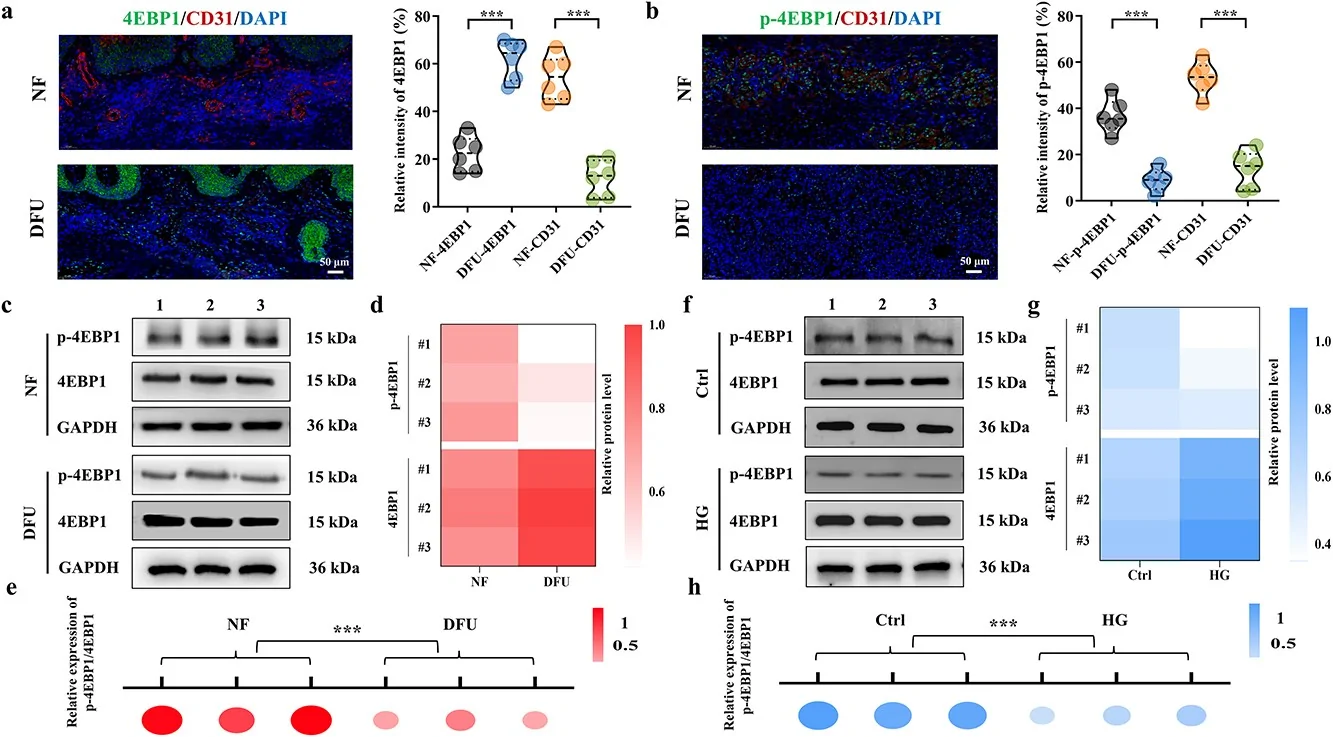
Experimental validation of p-4EBP1/4EBP1 dysregulation in DFU tissues and models. (**a**) Immunofluorescence staining and quantitative analysis of 4EBP1 and CD31 expression in histopathological sections of NF skin and periulcer skin from DFU patients. Scale bar: 50 μm. (**b**) Immunofluorescence staining and quantitative analysis of p-4EBP1 and CD31 expression in histopathological sections of NF skin and periulcer skin from DFU patients. Scale bar: 50 μm. (**c–e**) Western blot analysis and quantification of 4EBP1 phosphorylation levels in NF skin versus DFU periulcer skin tissues. (c) Representative western blot bands. (d and e) Statistical analysis of p-4EBP1 levels. (**f–h**) Western blot analysis and quantification of 4EBP1 phosphorylation levels in HDMECs under normal glucose conditions versus high-glucose conditions. (f) Representative western blot bands. (g and h) statistical analysis of p-4EBP1 levels. *n* = 3; ^***^P < 0.001. *CD31* platelet EC adhesion molecule-1, *p-4EBP1* phosphorylated 4EBP1, *DFU* diabetic foot ulcers, *NF* normal foot, *Ctrl* control, *HG* high glucose, *HDMECs* human dermal microvascular endothelial cells

To experimentally validate the dysregulation of p-4EBP1/4EBP1 identified in our single-cell transcriptomic analysis, we first performed immunofluorescence staining for 4EBP1 and the endothelial marker CD31 on histopathological sections of NF skin and periulcer skin from DFU patients. Representative IF images revealed a pronounced increase in 4EBP1 protein expression in the DFU group compared with that in the NF group, whereas the expression level of the endothelial marker CD31 and the phosphorylation level of 4EBP1 (p-4EBP1) were significantly reduced in DFU tissues (Figure 7a and b). We next investigated the functional state of 4EBP1 by assessing its phosphorylation level, which is a key regulatory mechanism. Western blot analysis of NF and DFU periulcer skin tissues revealed that p-4EBP1 expression was significantly lower in the DFU group than in the NF group (Figure 7c–e). To determine whether this dysregulation could be recapitulated *in vitro* under conditions mimicking diabetic hyperglycemia, we analysed p-4EBP1 levels in HDMECs. Consistent with the patient tissue findings, the results of the Western blot analysis revealed that compared with those cultured under normal glucose conditions, HDMECs cultured under high-glucose conditions presented significantly lower p-4EBP1 levels (Figure 7f–h). Collectively, these results confirm aberrant 4EBP1 expression and, critically, reduced phosphorylation in both DFU patient tissues and a cellular model of diabetic stress, substantiating its dysregulation in DFU pathogenesis.

### tdASC-EVs rescue hyperglycemia-induced suppression of PI3K/AKT/mTOR/4EBP1 signaling in HDMECs

**Figure 8:**
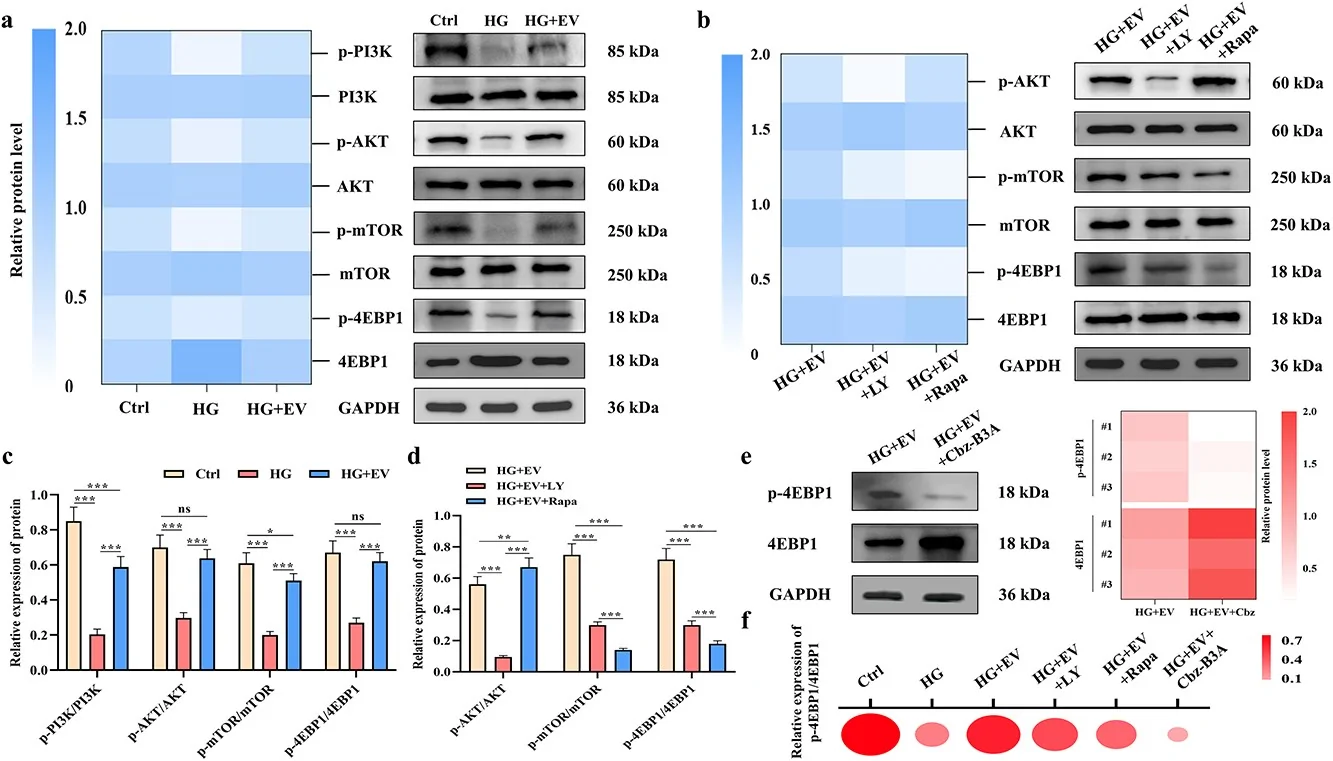
tdASC-EVs rescue hyperglycemia-induced suppression of PI3K/AKT/mTOR/4EBP1 signaling in HDMECs. (**a**) Western blot analysis of PI3K/AKT/mTOR/4EBP1 pathway activation in high-glucose-treated HDMECs with or without tdASC-EVs. (**b**) Effects of tdASC-EVs on PI3K/AKT/mTOR/4EBP1 signaling in HG–cultured HDMECs pretreated with the AKT inhibitor LY294002 (LY) or the mTOR inhibitor rapamycin (Rapa). (**c**) Results from the quantitative analysis of p-PI3K/PI3K, p-AKT/AKT, p-mTOR/mTOR, and p-4EBP1/4EBP1 ratios in HDMECs under high-glucose conditions with/without tdASC-EVs. (**d**) Statistical analysis of p-AKT/AKT, p-mTOR/mTOR, and p-4EBP1/4EBP1 ratios in HDMECs treated with tdASC-EVs following AKT or mTOR inhibition. (**e**) Western blot showing p-4EBP1/4EBP1 levels in high-glucose-cultured HDMECs treated with tdASC-EVs and a p-4EBP1-specific inhibitor. (**f**) Integrated quantification of p-4EBP1/4EBP1 ratios across all treatment groups. *n* = 3; ns, not significant; ^*^P < 0.5, ^**^P < 0.01, and ^***^P < 0.001. *HG* high glucose, *HDMECs* human dermal microvascular endothelial cells, *tdASC* three-dimensional adipose stem cell, *EV* extracellular vesicle, *AKT* Ak strain transforming

To investigate the regulatory role of tdASC-EVs in diabetic microvascular dysfunction, we evaluated their effects on the PI3K/AKT/mTOR/4EBP1 cascade in HDMECs (Figure 8). As shown in Figure 8a and c, exposure to high glucose (HG) markedly suppressed phosphorylation across this pathway, with significant reductions in p-PI3K/PI3K, p-AKT/AKT, p-mTOR/mTOR, and p-4EBP1/4EBP1 ratios (^***^P < 0.001 *vs* normal glucose control). Strikingly, tdASC-EV treatment significantly but partially restored these phosphorylation levels in HG-treated cells (^***^P < 0.001 *vs* HG alone), although these levels remained lower than those in normal controls. To elucidate the signaling hierarchy, we utilized targeted inhibitors (Figure 8b–d). Pretreatment with the AKT inhibitor LY294002 (LY) abolished tdASC-EV-induced AKT phosphorylation (^***^P < 0.001). mTOR inhibition by rapamycin (Rapa) effectively blocked mTOR autophosphorylation and downstream 4EBP1 phosphorylation (^***^P < 0.001 for all). Consistent with known feedback mechanisms, this inhibition was associated with a concomitant increase in AKT phosphorylation (Figure 8d). Consistent with the position of 4EBP1 downstream of mTOR, a p-4EBP1-specific inhibitor (Cbz-B3A) selectively attenuated tdASC-EV-mediated 4EBP1 phosphorylation under HG conditions (Figure 8e). Critically, integrated analysis of p-4EBP1/4EBP1 across all groups (Figure 8f) confirmed that tdASC-EVs specifically and significantly counteracted HG-induced 4EBP1 hypophosphorylation, collectively demonstrating that tdASC-EVs enhance the PI3K/AKT/mTOR/4EBP1 axis activity impaired by hyperglycemia.

### tdASC-EVs attenuate high-glucose-induced HDMEC senescence by promoting cap-dependent translation *via* increased p-4EBP1 levels

**Figure 9:**
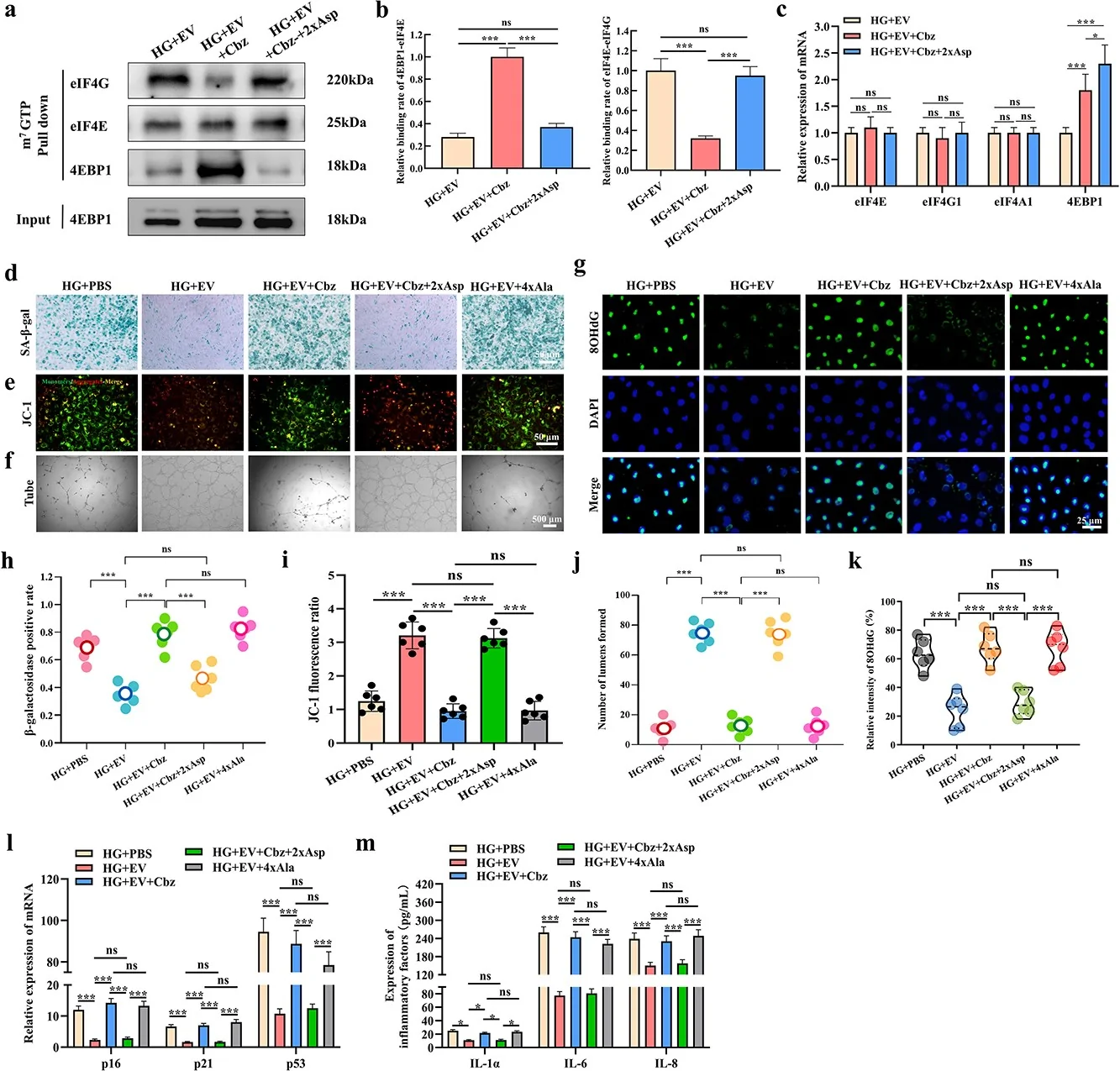
tdASC-EVs attenuate HDMEC senescence by increasing p-4EBP1 levels to facilitate the eIF4G– eIF4E interaction. (**a** and **b**) m^7^GTP pull-down assay and quantitative analysis of the association between 4EBP1 and eIF4E and between eIF4G and eIF4E following the inhibition or activation of p-4EBP1. (**c**) qRT–PCR analysis of the mRNA expression levels of cap-dependent translation-related genes (eIF4E, eIF4G, and eIF4A) across treatment groups. (**d**) Senescence-associated β-galactosidase (SA-β-Gal) staining. Scale bar: 50 μm. (**e**) Assessment of mitochondrial membrane potential using JC-1 staining. Scale bar: 50 μm. (**f**) Tube formation assay. Scale bar: 500 μm. (**g**) Measurement of 8-OHdG expression. Scale bar: 25 μm. (**h**) Quantitative analysis of SA-β-Gal staining. (**i**) Quantitative analysis of JC-1 staining. (**j**) Quantitative analysis of tube formation capacity. (**k**) Quantitative analysis of 8-OHdG expression. (**l**) Relative mRNA expression levels of senescence markers in different groups. (**m**) ELISA measurement of senescence-associated secretory phenotype (SASP) factor secretion. *n* = 6; ns, not significant; ^*^P < 0.5, ^**^P < 0.01, and ^***^P < 0.001. *tdASC* three-dimensional adipose stem cell, *EV* extracellular vesicle, HG high glucose, *HDMECs* human dermal microvascular endothelial cells, *SASP* senescence-associated secretory phenotype, *IL* interleukin, *PBS* phosphate-buffered saline

To further investigate the downstream mechanisms through which tdASC-EVs regulate p-4EBP1 levels and their functional implications, we inhibited (using Cbz-B3A) or activated (*via* transfection with 4EBP1-2xAsp) p-4EBP1 in high-glucose-cultured HDMECs treated with tdASC-EVs (Figure 9). m^7^GTP pull-down assays demonstrated that tdASC-EVs reduced the binding of 4EBP1 to eukaryotic initiation factor 4E (eIF4E) and increased the interaction between eukaryotic initiation factor 4G (eIF4G) and eIF4E; however, these effects were significantly diminished by p-4EBP1 inhibition. Importantly, transfection with the hyperphosphorylated 4EBP1 mutant (4EBP1-2xAsp) restored the ability of tdASC-EVs to decrease 4EBP1–eIF4E binding and increase eIF4G–eIF4E association (Figure 9a and b). The specificity of these interactions was underscored by control experiments, which revealed no detectable pull-down signal under either the bead-alone or the m^7^GDP competition conditions. To rule out transcriptional interference and confirm that the observed changes in eIF4E–eIF4G binding were driven by posttranslational competition rather than altered gene expression, we performed qPCR to examine the mRNA levels of key cap-dependent translation genes, including eIF4E, eIF4G, and eIF4A. The results indicated that neither inhibition nor activation of p-4EBP1 altered the transcriptional levels of these genes in tdASC-EV-treated HDMECs under high-glucose conditions, although p-4EBP1 inhibition led to the accumulation of the 4EBP1 protein, and its activation further increased transcription, thereby confirming the posttranslational origin of the results of the pull-down assay (Figure 9c). We subsequently evaluated HDMEC functionality under p-4EBP1 modulation (Figure 9d–m). tdASC-EV treatment significantly reduced high-glucose-induced senescence, as evidenced by decreased SA-β-galactosidase activity (Figure 9d and e), decreased mRNA expression of senescence markers (p16, p21, p53) (Figure 9f), and reduced SASP secretion (Figure 9l). Moreover, tdASC-EVs mitigated oxidative stress, as indicated by decreased 8-OHdG expression (Figure 9g and m), and restored the mitochondrial membrane potential, as measured by JC-1 staining (Figure 9h and i). Additionally, tdASC-EVs increased the impairment of the tube-forming capacity of HDMECs induced by HG (Figure 9j and k). Notably, these protective effects were abolished by p-4EBP1 inhibition. Collectively, these results demonstrate that tdASC-EVs increase p-4EBP1 levels to promote cap-dependent translation initiation, thereby alleviating cellular senescence and oxidative stress induced by high-glucose levels and improving angiogenic function in HDMECs.

### tdASC-EVs promote wound healing in diabetic mice by attenuating vascular senescence and increasing angiogenesis *via* p-4EBP1 signaling

**Figure 10:**
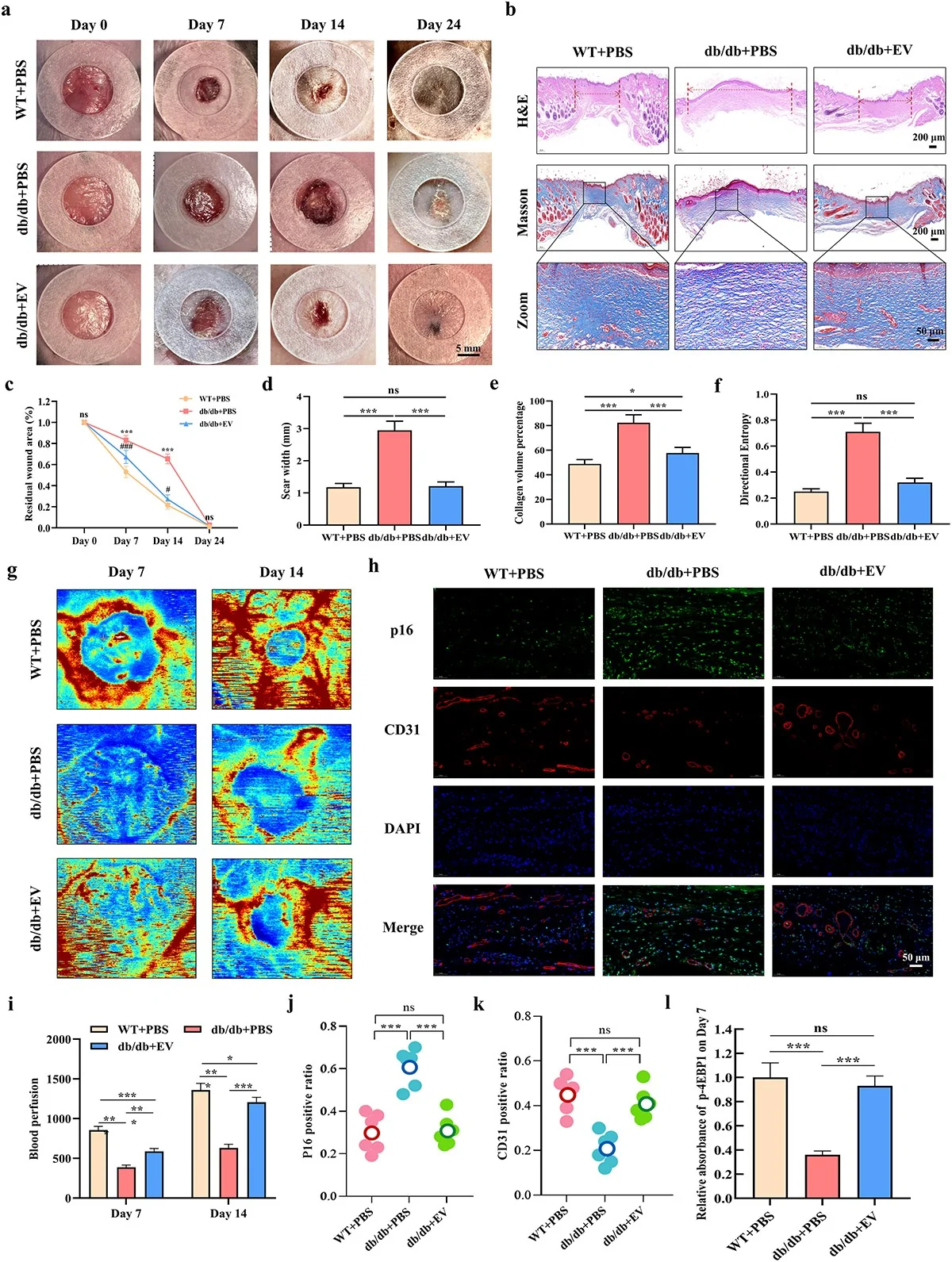
tdASC-EVs accelerate wound healing in diabetic mice by attenuating vascular senescence and promoting angiogenesis. (**a**) Representative images showing the wound healing process in diabetic mice treated with tdASC-EVs. Scale bar: 5 mm. (**b**) Histological evaluation of wound tissues across different groups via H&E and Masson staining. Scale bar: 50 μm and 200 μm. (**c**) Statistical analysis of the wound closure rates among the groups (^*^P < 0.5, ^***^P < 0.001 db/db + PBS *vs* WT + PBS; ^#^*P* < 0.5, ^###^*P* < 0.001 db/db + PBS *vs* db/db + EV). (d) The scar width was measured in H&E-stained sections. (**e**) Collagen volume fraction was quantified from Masson’s trichrome-stained sections. (**f**) Directional distribution entropy, a measure of collagen fiber alignment, was calculated from Masson’s trichrome-stained sections using a pixelwise fiber orientation analysis algorithm. (**g**) Blood perfusion around the wounds was measured by laser doppler imaging. (**h**) Immunofluorescence staining and quantitative analysis of p16 (a senescence marker) and CD31 (an endothelial marker) in periwound skin sections. Scale bar: 50 μm. (**i–k**) Statistical analysis of blood perfusion and the P16- and CD31-positive ratios. (**l**) Quantification of p-4EBP1 levels in periwound tissues by ELISA. *n* = 8; ns, not significant; ^*^P < 0.5, ^**^P < 0.01, and ^***^P < 0.001. *tdASC-EVs* tdASC-derived extracellular vesicles, *PBS* phosphate-buffered saline, *WT* wild-type, *EVs* extracellular vesicles, *H&E* haematoxylin and eosi, *ELISA* enzyme-linked immunosorbent assay

Building on our *in vitro* findings, we further investigated the proangiogenic and antisenescent effects of tdASC-EVs *in vivo* using a diabetic wound healing model. Full-thickness cutaneous wounds were created in WT and leptin receptor-deficient diabetic (db/db) mice, followed by local treatment with either PBS or tdASC-EVs (Figure 10). Compared with the WT + PBS group, the db/db + PBS group exhibited significantly delayed wound healing, whereas tdASC-EV treatment markedly accelerated the wound closure rate in the db/db + EV group (*P* < .5 or *P* < .01) (Figure 10a and c). Histological analysis of healed wounds *via* H&E and Masson’s staining revealed that compared with the WT controls, the db/db + PBS group presented increased scar width, disorganized collagen fiber arrangement, and reduced numbers of skin appendages and large vessels. In contrast, tdASC-EV treatment significantly reduced scar width, restored organized collagen deposition, and increased the abundance of skin appendages and large vessels in db/db + EV mice (Figure 10b–e). More importantly, EV treatment markedly improved collagen fiber alignment, as reflected by a significant reduction in the directional distribution entropy calculated from Masson’s trichrome-stained sections using a validated pixelwise fiber orientation algorithm (Figure 10f), suggesting a shift from disorganized fibrotic deposition toward a more orderly, scar-less remodeling pattern. Laser Doppler imaging demonstrated that compared with that in the WT + PBS group, blood perfusion around the wound was substantially impaired in the db/db + PBS group at Days 7 and 14, and this deficit was significantly ameliorated by tdASC-EV administration (Figure 10g and i). Furthermore, immunofluorescence staining of periwound skin on Day 14 revealed that the expression of the senescence marker p16 was significantly upregulated, whereas the expression of the endothelial marker CD31 was downregulated in the db/db + PBS group compared with that in the WT control group. tdASC-EV treatment notably reduced p16 expression and increased CD31 expression in db/db + EV mice (Figure 10h, j, and  k). Additionally, the ELISA results indicated that p-4EBP1 levels in the periwound tissue on Day 7 were significantly lower in the db/db + PBS group than in the WT control group, and tdASC-EV treatment effectively restored p-4EBP1 expression in the db/db + EV group (Figure 10l). Taken together, these results demonstrate that tdASC-EVs promote diabetic wound healing *in vivo* by increasing p-4EBP1 expression, attenuating vascular senescence, and facilitating functional angiogenesis.

### Proteomic profiling reveals a proregenerative protein cargo enriched in tdASC-EVs

**Figure 11:**
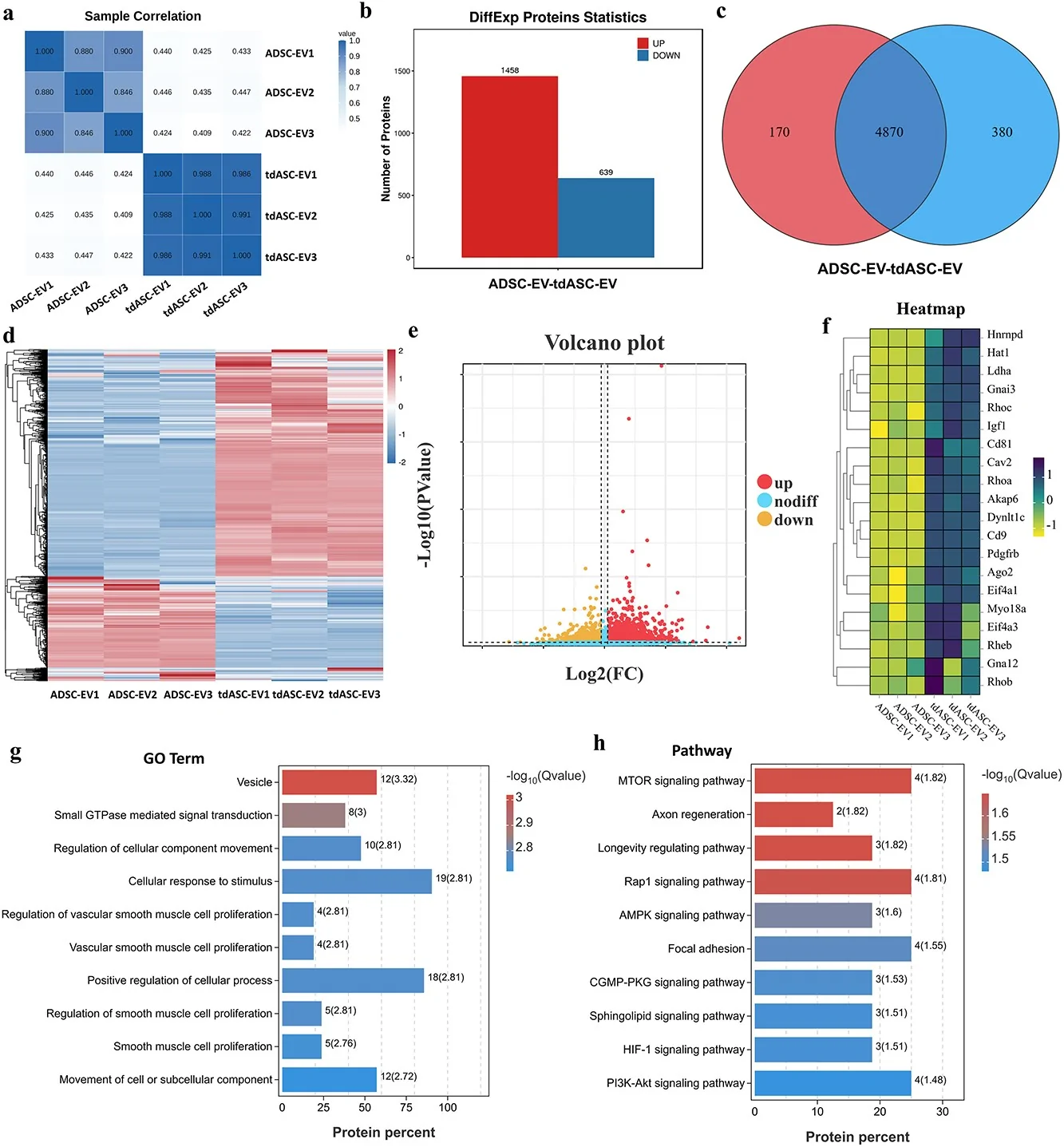
Proteomic analysis revealed a functionally enriched protein profile in tdASC-EVs. (**a**) Pearson correlation heatmap of all samples based on global protein expression intensity, which revealed high intragroup reproducibility. (**b**) Bar graph showing the total number of DEPs between tdASC-EVs and ADSC-EVs (FC >2, *P* < 0.05). (**c**) Venn diagram illustrating the overlap of DEPs between the two groups, revealing proteins common to both groups and those unique to tdASC-EVs. (**d**) Heatmap of hierarchical clustering analysis for all DEPs. Each column represents an individual sample, and each row represents a protein. The expression levels are normalized to the Z score and color coded (red, high expression; blue, low expression). (**e**) Volcano plot of the DEPs. Significantly upregulated (1458) and downregulated (639) proteins are highlighted in red and blue, respectively (FC >2, *P* < .05). Dashed lines indicate the thresholds. (**f**) Heatmap displaying the relative expression levels of 20 key upregulated proteins of interest selected for their relevance to proregenerative mechanisms. (**g**) Top 10 enriched GO biological process terms for the 20 candidate proteins. (**h**) Top 10 enriched KEGG pathways for the 20 candidate proteins. *tdASC-EVs* tdASC-derived extracellular vesicles, *ADSC-EVs* adipose-derived mesenchymal stem cell-extracellular vesicles

To elucidate the molecular basis underlying the enhanced therapeutic efficacy of tdASC-EVs, we performed a comparative proteomic analysis of EVs derived from tdASCs and conventional ADSCs. Unsupervised hierarchical clustering of all samples based on global protein expression showed excellent intragroup reproducibility (Figure 11a). We identified 2097 DEPs (FC >2, *P* < .05), with 1458 significantly upregulated and 639 downregulated in tdASC-EVs (Figure 11b). A Venn diagram illustrated the distribution of these DEPs, with 4870 proteins commonly detected in both groups and 380 unique DEPs present specifically in tdASC-EVs (Figure 11c). A heatmap of all the DEPs revealed a clear segregation between the two EV groups (Figure 11d), which was further visualized in a volcano plot highlighting the proteins with the most significant changes in expression (Figure 11e). From this extensive dataset, we focused on 20 highly upregulated proteins on the basis of their established roles in proregenerative processes. This curated panel, which included pivotal regulators of the PI3K–AKT–mTOR axis (IGF1, PDGFRB, and RHEB), cap-dependent translation (eIF4A1/3), cytoskeletal dynamics (Rhoa/b/c and Gna12/13), and epigenetic regulation (Hat1), is presented in a focused heatmap (Figure 11f). GO enrichment analysis of these 20 proteins confirmed their collective involvement in key repair-related biological processes, such as small GTPase-mediated signal transduction and the regulation of vascular smooth muscle cell proliferation (Figure 11g). Most compellingly, KEGG pathway analysis revealed significant enrichment in the “PI3K–Akt signalling pathway” and the “mTOR signalling pathway”, directly aligning with our proposed mechanistic framework (Figure 11h). Collectively, these proteomic data demonstrate that tdASC-EVs are selectively packaged with a functionally coherent protein cargo poised to activate proregenerative signaling and counteract pathological barriers, thereby providing a molecular rationale for their superior therapeutic performance.

### Large animal validation confirms the therapeutic efficacy of tdASC-EVs in diabetic wound healing

**Figure 12:**
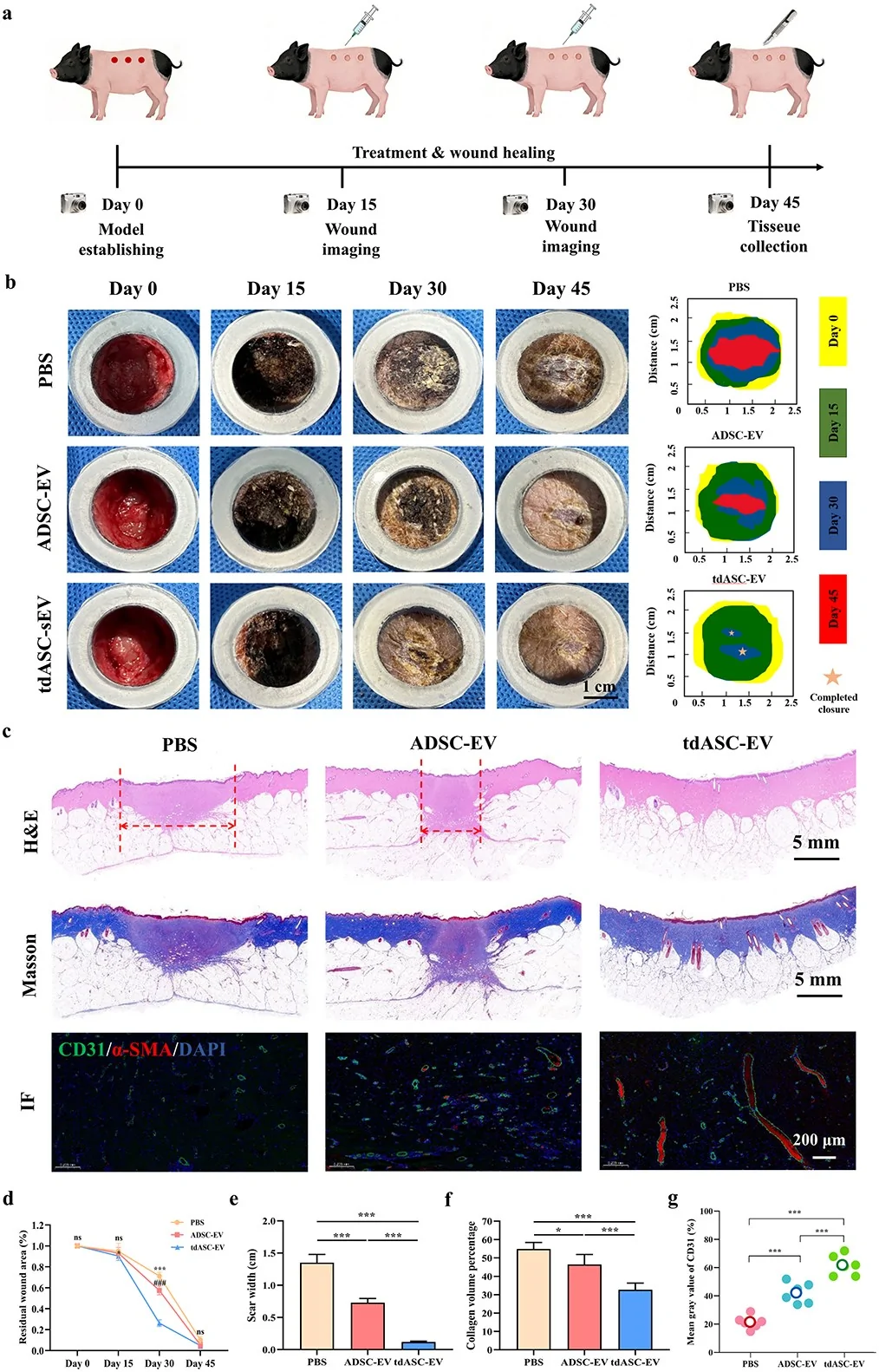
Therapeutic evaluation of ADSC-EVs and tdASC-EVs in a diabetic minipig wound model. (**a**) Gross image showing the creation of full-thickness cutaneous wounds (2 cm in diameter) on the back of a Bama minipig with induced diabetes. (**b**) Representative gross images of wounds from the PBS, ADSC-EV, and tdASC-EV treatment groups at the indicated time points post-wounding. Scale bar: 1 cm. (**c**) Representative histological and immunofluorescence images of healed wound tissues on Day 45. From top to bottom: H&E staining, Masson’s trichrome staining, and immunofluorescence staining for CD31 and α-SMA. Scale bars: H&E and Masson staining, 5 mm; immunofluorescence staining, 200 μm. (**d**) Quantitative analysis of wound closure. The percentage of remaining wound area relative to the original area was plotted over time for each group. PBS vs. tdASC-EVs, ^*^P < 0.05 and ^***^P < 0.001; ADSC-EVs *vs* tdASC-EVs, ###*P* <0.001. (**e**) Quantitative analysis of scar width in the healed tissues from each group. (**f**) Quantitative analysis of the percentage of collagen deposition in the dermal region of healed tissues, as measured from Masson’s trichrome-stained sections. (**g**) Quantitative analysis of neovascularization, represented by the mean fluorescence intensity of CD31 staining in the wound bed. *n* = 8, ^***^P < 0.001. *tdASC-EVs* tdASC-derived extracellular vesicles, *ADSC-EVs* adipose-derived mesenchymal stem cell-extracellular vesicles, *PBS* phosphate-buffered saline, *tdASCs* three-dimensional adipose stem cells

To rigorously evaluate and compare the therapeutic efficacy of ADSC-EVs and tdASC-EVs in a clinically relevant model, we established full-thickness cutaneous wounds on the backs of Bama minipigs with induced diabetes (Figure 12). Wounds were treated with equal doses of tdASC-EVs, ADSC-EVs, or PBS (control). Gross morphological observation over a 45-day period revealed markedly accelerated wound closure in the tdASC-EV group compared with that in both the ADSC-EV group and the PBS group (Figure 12a and b). Quantification of the remaining wound area confirmed this observation: wounds treated with tdASC-EVs healed significantly faster, with a statistically significant reduction in wound area compared with that of ADSC-EV-treated wounds on Day 15 (*P* < .05) and Day 30 (*P* < .001) (Figure 12d). Histopathological and immunohistochemical analyses of healed tissues at the endpoint provided deeper insights into the quality of regeneration. H&E, Masson’s trichrome, and immunofluorescence staining demonstrated that tdASC-EV treatment promoted the most complete regeneration of tissue architecture, characterized by robust regeneration of skin appendages, mature neovascularization, and minimal scar formation (Figure 12c). In contrast, ADSC-EV-treated wounds, while fully re-epithelialized, exhibited incomplete dermal regeneration, residual inflammatory cell infiltration, and noticeable scar tissue, albeit with substantial neovessel formation. The poorest outcomes were observed in the PBS-treated wounds, which exhibited wide scars, prominent inflammation, and only a small number of new vessels (Figure 12c). Morphometric analyses substantiated these qualitative findings. The scar width in the tdASC-EV group was significantly smaller than that in both the ADSC-EV and PBS groups (*P* < .001) (Figure 12e). Furthermore, collagen deposition, as assessed by Masson’s trichrome staining, was significantly lower in the tdASC-EV group, indicating a more regenerative (rather than fibrotic) healing pattern (Figure 12f). Finally, quantification of CD31 immunofluorescence staining intensity revealed that tdASC-EV treatment resulted in the greatest degree of neovascularization, significantly exceeding that in the ADSC-EV and PBS groups (*P* < .001) (Figure 12g). Collectively, these *in vivo* data from a large, diabetic animal model unequivocally demonstrate that tdASC-EVs are superior to conventional ADSC-EVs in promoting not only the speed but also, more importantly, the quality of wound healing by increasing vascularization and directing the process toward regeneration with reduced fibrosis.

## Discussion

DFUs remain a devastating complication of diabetes. They are characterized by impaired wound healing and high amputation rates. While multiple factors contribute to its pathogenesis, EC dysfunction is increasingly recognized as a central player in impaired angiogenesis and tissue repair in diabetes [8, 20]. Our single-cell transcriptomic analysis provides compelling evidence that HDMECs from DFU patients undergo significant phenotypic alterations and exhibit a pronounced senescent state. This dysfunction is accompanied by dysregulation of the PI3K/AKT/mTOR/4EBP1 signaling axis—a critical pathway that governs cellular translation, survival, and metabolism [21, 22]. These findings implicate endothelial senescence as a central mechanism in DFU pathology and suggest that targeting this process may offer therapeutic benefits.

The SFL-3D culture system effectively promoted the formation of tdASC spheroids with increased expression of ADSC markers (CD90 and OCT4) and differentiation potential, which was consistent with the findings of previous studies showing that 3D culture environments better mimic the native stem cell niche and promote the retention of primitive properties [9]. However, while prior research has largely focused on the differentiation and proliferation capacities of 3D-cultured MSCs, our study extends these findings by systematically evaluating the paracrine function—particularly EV secretion—of tdASCs. We found that compared with their 2D-cultured counterparts, tdASCs possess greater EV biogenesis capacity, which may explain their superior therapeutic efficacy.

In parallel, cell-assisted lipotransfer has emerged as a promising strategy for soft tissue reconstruction, but its efficacy in diabetic patients remains suboptimal because of poor graft vascularization and survival [23–25]. Conventional ADSCs often exhibit reduced functionality under standard 2D culture conditions, limiting their regenerative potential. Our study introduced a novel strategy in which SFL-3D culture was used to generate tdASCs with enhanced stemness and secretory properties. We demonstrated that tdASCs significantly improved fat graft retention and vascularization *in vivo*, effects that we hypothesized to be mediated largely through their secreted tdASC-EVs. Building on evidence that MSC-EVs can promote angiogenesis and mitigate cellular senescence [26, 27], we further investigated whether tdASC-EVs could reverse high-glucose-induced dysfunction in the endothelial compartment and facilitate diabetic wound healing.

Single-cell transcriptomic profiling of DFU and normal skin tissues revealed significant dysregulation within the endothelial compartment, specifically highlighting a shift in microvascular endothelial subpopulations and a pronounced senescent phenotype in HDMECs. Although previous studies on DFU have largely relied on ECs or human umbilical vein endothelial cells (HUVECs), these models differ considerably from HDMECs in terms of both phenotype and function. Our data underscore that HDMECs—rather than HUVECs or generic ECs—are central players in DFU pathogenesis. Through a series of *in vitro* experiments, we confirmed that tdASC-EVs effectively alleviated high-glucose-induced senescence, reduced oxidative stress, restored the mitochondrial membrane potential, and increased the tube formation capacity of HDMECs.

Notably, pathway analysis revealed that the PI3K/AKT/mTOR/4EBP1 axis is closely associated with translational control and cellular senescence. Importantly, 4EBP1 emerged as the sole gene with significantly upregulated expression involved in translation initiation in the HDMEC of DFU patients, and regulatory network analysis revealed that 4EBP1 is involved in the PI3K/AKT signaling cascade. Therefore, we focused on the PI3K/AKT/mTOR/4EBP pathway to investigate the mechanistic role of tdASC-EVs in modulating HDMEC senescence and promoting diabetic wound healing. Our results revealed significant upregulation of total 4EBP1 protein expression in DFU tissues compared with that in normal skin and a concurrent decrease in CD31^+^ microvascular density. These findings validated our single-cell sequencing data and suggested a seemingly paradoxical but not uncommon scenario in cellular signaling: increased total 4EBP1 coupled with decreased phosphorylation (p-4EBP1) [28, 29]. We propose that the increase in 4EBP1 expression may represent a compensatory transcriptional response or altered protein stability secondary to the chronic inhibition of upstream PI3K/AKT/mTOR signaling in the diabetic microenvironment. This pathway inhibition is directly reflected in the significant reduction in the levels of p-4EBP1, the functionally active form that dissociates from eIF4E to permit cap-dependent translation initiation. During this process, eIF4E recognizes and binds to the 5′-cap structure of mRNAs, recruiting other initiation factors, such as eIF4G, to assemble the translation initiation complex. Under DFU conditions, hypophosphorylated 4EBP1 binds to and sequesters eIF4E, preventing its interaction with eIF4G and thereby inhibiting cap-dependent translation; phosphorylation of 4EBP1 results in the release of eIF4E, allowing the formation of the eIF4E–eIF4G complex and facilitating the recruitment of ribosomes to mRNA. Consequently, the critical functional deficit is not only the abundance of 4EBP1 but also its inadequate phosphorylation, which leads to sustained translational repression. This rationale precisely guided our subsequent focus on investigating the restoration of p-4EBP1 expression by tdASC-EVs rather than total 4EBP1 expression. Our mechanistic studies confirmed that tdASC-EVs specifically target and reactivate the upstream PI3K/AKT/mTOR axis, thereby increasing p-4EBP1 levels, alleviating its inhibitory binding to eIF4E, and ultimately rescuing cap-dependent translation and cellular function in HDMECs. Using specific pharmacological inhibitors, namely, LY294002 (AKT inhibitor), rapamycin (mTOR inhibitor), and Cbz-B3A (p-4EBP1 inhibitor), we systematically characterized the signaling hierarchy and established that tdASC-EVs act through this axis to exert their cytoprotective effects. Importantly, our m^7^GTP pull-down assays revealed that tdASC-EVs promote the dissociation of 4EBP1 from eIF4E and facilitate the assembly of the eIF4E–eIF4G translation initiation complex, thereby increasing cap-dependent translation. This mechanism is critical for maintaining proteostasis and cellular homeostasis under stress conditions [30]. Subsequent rescue experiments using a hyperphosphorylated 4EBP1 mutant (4EBP1-2xAsp) further substantiated that tdASC-EV-mediated preservation of cap-dependent translation is essential for mitigating endothelial senescence and dysfunction. Furthermore, proteomic sequencing of tdASC-EVs revealed the enrichment of numerous proteins that activate this pathway. The SFL-3D culture method, by collectively enriching EVs with these synergistic proteins, effectively initiates and sustains the proregenerative signaling cascade. These results provide a mechanistic depth that was previously lacking in studies focusing solely on the phenotypic outcomes of EV-based therapies. To our knowledge, this study provides the first high-resolution single-cell atlas highlighting the contribution of HDMEC senescence to impaired wound repair in DFUs and establishes 4EBP1 as a novel therapeutic target specifically altered in the DFU-associated microvascular endothelium.

Building on this mechanistic understanding, it is instructive to contextualize the therapeutic potential of tdASC-EVs within the broader landscape of MSC-derived EV therapies. In the broader landscape of MSC-EV therapies for diabetic wounds, our findings concerning tdASC-EVs invite a comparative perspective with findings concerning EVs from other prominent sources, such as bone marrow (BMSC-EVs) or umbilical cord (UCSC-EVs). Each source has distinct practical and biological characteristics. ADSCs, and by extension tdASCs, are typically obtained from elective liposuction procedures, offering a highly accessible and abundant source with fewer ethical constraints than BMSCs or UCSCs. It is often reported that ADSC-EVs are particularly enriched in proangiogenic factors, which aligns with our observed potent effects on HDMEC function and wound vascularization. In contrast, UCSC-EVs may exhibit superior immunomodulatory properties, which could be advantageous in highly inflammatory wound environments. The key advancement presented here is not merely the adipose origin but also the significant functional amplification achieved through the SFL-3D culture system, which sets tdASC-EVs apart from conventionally cultured ADSC-EVs. These findings underscore the principle that both the cellular source and the culture strategy are critical determinants of the therapeutic potency of EVs.

*In vivo*, tdASC-EVs not only enhanced fat graft survival and vascularization but also significantly accelerated wound closure in diabetic mice. These benefits were associated with increased CD31^+^ microvessel density, reduced expression of the senescence marker p16, and restored p-4EBP1 levels in wound tissues. Notably, the ~20% improvement in fat graft retention induced by tdASC-EVs, although seemingly modest in numerical value, is substantially clinically significant for diabetic patients. In clinical practice, fat grafts in diabetic individuals suffer from an extremely high absorption rate (40%–60%) because of impaired angiogenesis, chronic inflammation, and endothelial dysfunction, which leads to frequent secondary procedures and poor patient outcomes. A 20% increase in graft retention can effectively reduce the need for revision surgeries, lower medical costs, and improve long-term esthetic and functional outcomes for diabetic patients undergoing soft tissue reconstruction. Furthermore, this therapeutic advantage was further validated in our diabetic Bama miniature pig model, a clinically relevant large animal system that recapitulates human diabetic wound pathophysiology. Compared with conventional ADSC-EVs, tdASC-EVs not only enhanced fat graft survival but also accelerated wound closure, promoted neovascularization, and reduced scarring, indicating that tdASC-EVs are a multifunctional acellular therapeutic agent. Unlike single-effect EV-based therapies currently under preclinical investigation, tdASC-EVs address multiple pathological barriers in diabetic wounds (endothelial senescence, insufficient angiogenesis, and fibrotic scarring) through a unified mechanism, thereby exhibiting superior translational potential over existing EV therapies. Our findings align with emerging evidence that MSC-EVs can modulate cellular senescence and protein synthesis pathways [8]. However, given the confounding effects associated with the administration of pathway inhibitors in diabetic wound models, *in vivo* rescue experiments were not performed in this study. Future validation using conditional gene knockout models is warranted. Nevertheless, this is the first report to show that the ability of EVs derived from SFL-3D-cultured MSCs to regulate the translational machinery under diabetic conditions is enhanced. To directly address the gap between standard rodent models and human clinical application, we conducted a supportive pilot study in a porcine diabetic full-thickness wound model. This large animal model shares significant physiological and healing kinetic similarities with human skin and provides a more clinically relevant bridge than rodent studies alone.

Despite these promising findings, our study has several limitations. First, the single-cell RNA sequencing analysis, while insightful, was based on a relatively limited sample size. Future studies with larger cohorts are warranted to validate the endothelial subpopulation dynamics and transcriptional alterations identified here. Second, although our proteomic analysis revealed multiple key proteins enriched in tdASC-EVs that are involved in activating the PI3K/AKT/mTOR/4EBP1 axis, including IGF1, PDGFRB, RHEB, and eIF4A1/3, which are theoretically capable of initiating the phosphorylation cascade of PI3K, AKT, mTOR, and 4EBP1 in HDMECs, the specific functional roles of these individual protein cargoes have not been directly verified by loss- or gain-of-function experiments (protein neutralization, gene silencing, or ectopic expression) in the present study. These candidate proteins are hypothesized to be delivered into HDMECs via tdASC-EV internalization, where they directly trigger the phosphorylation of PI3K and sequentially activate the downstream AKT/mTOR/4EBP1 signaling module, thereby alleviating 4EBP1-mediated translational repression and restoring cap-dependent translation. In future studies, we will perform targeted rescue assays, antibody-mediated neutralization, and CRISPR/Cas9 gene editing to confirm the core effector proteins in tdASC-EVs that drive the activation of the PI3K/AKT/mTOR/4EBP1 pathway and mediate the anti-senescent and pro-angiogenic effects on diabetic microvascular ECs. This follow-up work will further clarify the precise molecular mechanism through which tdASC-EVs treat diabetic wounds. Additionally, although we demonstrated increased translational regulation by SFL-3D-derived exosomes under diabetic conditions, the interplay between endothelial senescence and apoptosis—both linked to this pathway—remains unexplored. Emerging evidence suggests that these processes share molecular regulators (TERF2IP and p53/p21) and that senescent cells exhibit altered apoptotic thresholds under diabetic stress [31, 32]. Future studies using endothelial-specific conditional knockout models are warranted to dissect this axis and validate pathway involvement without confounding systemic effects. Beyond preclinical efficacy, the clinical translation of tdASC-EVs necessitates rigorous long-term safety assessments. Future investigational new drug-enabling studies should comprehensively evaluate their biodistribution and clearance kinetics, immunogenicity profiles, including acute reactions and anti-EV antibody development, and potential protumorigenic effects in susceptible models. Addressing these points will be essential for establishing a therapeutic window and advancing tdASC-EVs toward clinical application for treating diabetic complications.

## Conclusions

In summary, our study revealed that tdASC-EVs derived from SFL-3D-cultured ADSCs promote diabetic wound healing by alleviating microvascular EC senescence through activation of the PI3K/AKT/mTOR/4EBP1 signaling pathway and enhancement of cap-dependent translation. These findings not only provide a novel strategy for improving MSC-EV functionality but also highlight the critical role of protein translational regulation in diabetic wound pathogenesis. This work supports the future development of tdASC-EVs as a promising acellular therapy for diabetic complications.

## Abbreviations

ALP: alkaline phosphatase; ADSCs: adipose-derived mesenchymal stem cells; ADSC-EVs: adipose-derived mesenchymal stem cell-extracellular vesicles; AKT: Ak strain transforming; DAPI: 4′6-diamidino-2-phenylindole; DFU: diabetic foot ulcers; DMEM: Dulbecco’s modified Eagle’s medium; ELISA: enzyme-linked immunosorbent assay; EVs: extracellular vesicles; ECs: endothelial cells; FBS: fetal bovine serum; FC: fold change; HDMECs: human dermal microvascular endothelial cells; H&E: hematoxylin and eosin; HUVECs: human umbilical vein endothelial cells; HG: high glucose; HRP: horseradish peroxidase; KEGG: Kyoto Encyclopedia of Genes and Genomes; KLF4: Krüppel-like factor 4; IGF-1: insulin-like growth factor 1; IHC: immunohistochemical; LY: LY294002; MSCs: mesenchymal stem cells; mTOR: mechanistic target of rapamycin; NF: normal foot; OCT4: octamer-binding transcription factor 4; OD: optical density; PBS, phosphate-buffered saline; PI3K: phosphatidylinositol 3-kinase; PPARγ: peroxisome proliferator-activated receptor gamma; PDGF: platelet-derived growth factor; PE: phycoerythrin; qRT–PCR: quantitative reverse transcription-polymerase chain reaction; Rapa: rapamycin; ROI: region of interest; SASP: senescence-associated secretory phenotype; SD: standard deviation; SFL-3D: self-feeder layer 3D; TEM: transmission electron microscopy; TSG101: tumor susceptibility gene 101; tdASCs: three-dimensional adipose stem cells; tdASC-EVs: tdASC-derived extracellular vesicles; UTR: untranslated region; 8OHdG: 8-hydroxy-2′-deoxyguanosine; 4EBP1: 4E-binding protein 1; p-4EBP1: phosphorylated-4EBP1.

## Supplementary Material

Supplementary_material_tkag042

## Data Availability

The data used to support the findings of this study are available from the corresponding author upon request.
