# Supplementary material for: SFL-3D-cultured adipose mesenchymal cell-derived extracellular vesicles promote diabetic wound healing by alleviating microvascular endothelial senescence via PI3K/AKT/mTOR/4EBP1-mediated cap-dependent translation
Source: Burns Trauma. 2026 Jun 22;14:tkag042. doi: 10.1093/burnst/tkag042 (PMC13571603; doi:10.1093/burnst/tkag042)
Supplement: Supplementary file 1 [file supplementary_material_tkag042.zip › Figure S1.docx]

**Supplementary**

**Figure S1.** Comparison of EV secretion ability and EV yield between ADSCs and tdASCs. (**a**) Expression of genes involved in EV biogenesis and secretion pathways in ADSCs and tdASCs determined by qRT‒PCR (*n* = 4). (**b**) Identification of EV markers by Western blotting. (**c**) Distribution determined from the TEM images. (**d**) EV production from ADSC and tdASC cultures at 2-day intervals was determined by nanoparticle tracking analysis (NTA) and normalized to the cell number on day 2 (*n* = 6). (**e**) Total protein quantification in ADSC-EVs and tdASC-EVs (*n* = 5). **p* < 0.5, ***p* < 0.01, and ****p* < 0.001. *EV* extracellular vesicle, *ADSC* adipose-derived mesenchymal stem cells, *tdASC* three-dimensional adipose stem cells, *tdASC-EV* tdASC-derived extracellular vesicles.
