# Supplementary material for: SFL-3D-cultured adipose mesenchymal cell-derived extracellular vesicles promote diabetic wound healing by alleviating microvascular endothelial senescence via PI3K/AKT/mTOR/4EBP1-mediated cap-dependent translation
Source: Burns Trauma. 2026 Jun 22;14:tkag042. doi: 10.1093/burnst/tkag042 (PMC13571603; doi:10.1093/burnst/tkag042)
Supplement: Supplementary file 1 [file supplementary_material_tkag042.zip › S1-S2_tkag042.docx]

**Supplementary**

**Figure S1.** Comparison of EV secretion ability and EV yield between ADSCs and tdASCs. (a) Expression of genes involved in EV biogenesis and secretion pathways in ADSCs and tdASCs determined by qRT‒PCR (*n* = 4). (b) Identification of EV markers by Western blotting. (c) Distribution determined from the TEM images. (d) EV production from ADSC and tdASC cultures at 2-day intervals was determined by nanoparticle tracking analysis (NTA) and normalized to the cell number on day 2 (*n* = 6). (e) Total protein quantification in ADSC-EVs and tdASC-EVs (*n* = 5). **p* < 0.5, ***p* < 0.01, and ****p* < 0.001. EV, extracellular vesicle; ADSC, adipose-derived mesenchymal stem cells; tdASC, three-dimensional adipose stem cells; tdASC-EV, tdASC-derived extracellular vesicles.

**Figure S2.** Comparison of the ability of ADSC-EVs and tdASC-EVs to promote lipogenesis and angiogenesis in vivo. (a) Ultrastructures of ADSC-EVs visualized via transmission electron microscopy (TEM). Scale bar: 100 nm. (b) Particle size distribution of tdASC-EVs determined using NanoFCM. (c–d) Anatomical images 4 weeks after the transplantation of the granular fat into the nude mice. and statistical analysis of retention quality after fat transplantation in the four groups. G1 represents the simple granular fat transplantation group, G2 represents the ADSC mixed granular fat transplantation group, G3 represents the tdASC-EV mixed granular fat transplantation group, and G4 represents the ADSC-EV mixed granular fat transplantation group. (e–g) Statistical analysis of three-dimensional CT images and retained volumes of the four groups after fat transplantation. (h–j) Immunofluorescence staining and statistical analysis of lipogenic markers and angiogenesis markers in tissue sections from each group after fat transplantation. *n*=8; **p* < 0.5, ***p* < 0.01, and ****p* < 0.001.

ADSC-EVs, ADSC-derived extracellular vesicles; tdASC-EVs, tdASC-derived extracellular vesicles.
