# Supplementary material for: SFL-3D-cultured adipose mesenchymal cell-derived extracellular vesicles promote diabetic wound healing by alleviating microvascular endothelial senescence via PI3K/AKT/mTOR/4EBP1-mediated cap-dependent translation
Source: Burns Trauma. 2026 Jun 22;14:tkag042. doi: 10.1093/burnst/tkag042 (PMC13571603; doi:10.1093/burnst/tkag042)
Supplement: Supplementary file 1 [file supplementary_material_tkag042.zip › Supplementary_Table_S1new_tkag042(1).docx]

| **Gene Symbol** | **Forward Primer**  **(5‘→3’)** | **Reverse Primer**  **(5‘→3’)** | **Amplicon Size**  **(bp)** | **NCBI Primer-BLAST Specificity Summary** | **Reference for Primer Sequence** |
| --- | --- | --- | --- | --- | --- |
| KLF4 | TCTCTTCGTGCACCCACTTG | GGCATGAGCTCTTGGTAATGG | 145 | [Unique product for human KLF4 (RefSeq: NM_004235.6)](https://www.ncbi.nlm.nih.gov/entrez/viewer.fcgi?db=nucleotide&id=1519314269) | Tan, Yiling et al. J Transl Med. 2024. |
| ALP | AGTGCTCTGCGCAGGATTG | CGCCAGTACTTGGGGTCTTT | 207 | [Unique product for human ALPL (RefSeq: NM_000478.6)](https://www.ncbi.nlm.nih.gov/entrez/viewer.fcgi?db=nucleotide&id=1519314269) | Martínez-Heredia, Luis et al. Front Endocrinol (Lausanne). 2024. |
| Nanog | CCTGTGATTTGTGGGCCTG | GACAGTCTCCGTGTGAGGCAT | 78 | Unique product for human Nanog (RefSeq: NM_024865.4) | Kainulainen, Kirsi et al. Oncoimmunology. 2024. |
| Sox2 | GTATCAGGAGTTGTCAAGGCAGAG | TCCTAGTCTTAAAGAGGCAGCAAAC | 78 | Unique product for human Sox2 (RefSeq: NM_003106.4) | Kainulainen, Kirsi et al. Oncoimmunology. 2024. |
| CD9 | ATGCTGGGACTGTTCTTCGG | GAATATCCCCAGATGGCCGC | 74 | Unique product for human CD9 (RefSeq: NM_001769.4) | Ondruššek, R et al.Klin Onkol. 2024 |
| CD63 | ACAACCACACTGCTTCGATCC | GACTCGGTTCTTCGACATGGA | 110 | Unique product for human CD63 (RefSeq: NM_001413282.1) | Yuan X, et al. J Extracell Vesicles. 2022. |
| CD81 | ATCCTGTTTGCCTGTGAGGTG | TGCTGTAGGGCCTGGTCATAG | 101 | Unique product for human CD81 (RefSeq: NM_001425131.1) | Yuan X, et al. J Extracell Vesicles. 2022. |
| Alix | TAAGTGCATCTGAGGGCCAAA | GGGGCCTCCTTTCCTAGTTTC | 112 | Unique product for human PDCD6IP (RefSeq: XM_047447042.1) | Yuan X, et al. J Extracell Vesicles. 2022. |
| TSG101 | CACCTGGTGGTCCATATCCTG | GATGGTGTCCTCGCTGATTGT | 107 | Unique product for human TSG101 (RefSeq: XM_005253108.5) | Yuan X, et al. J Extracell Vesicles. 2022. |
| HRS | TGATGACCCGTAAGCACCAC | CTTTCCCTCCGCTTTCCTGT | 155 | Unique product for human HGS (RefSeq: NM_004712.5) | Gonzalez-Lozano, Miguel A et al. Nature. 2025. |
| Syntenin | TTATTAAGCGGATGGCACCAA | TTGCCAAAGAAGGAAACTGGA | 120 | Unique product for human SDCBP (RefSeq: NM_001007069.2) | Yuan X, et al. J Extracell Vesicles. 2022. |
| Rab27B | TAAGTAGCTGTCCCCGTGCT | TTTGTCAGCCAGTTCCCGAG | 631 | Unique product for human RAB27B (RefSeq: NM_004163.4) | Schaffer, Leah V et al. Nature. 2025. |
| Rab27A | GCATGTTTCAGTTTTCAAGAACCA | AAAGGTGGCTTTTGTGTGTGC | 110 | Unique product for human Rab27A (RefSeq: NM_001438970.1) | Yuan X, et al. J Extracell Vesicles. 2022. |
| ISG15 | CTCTGAGCATCCTGGTGAGGAA | AAGGTCAGCCAGAACAGGTCGT | 136 | Unique product for human ISG15 (RefSeq: NM_005101.4) | Yuan X, et al. J Extracell Vesicles. 2022. |
| Flotillin1 | CAGGAAAGCTGGTCTGCGAG | TGCTTCTCCAATCCGTGCAT | 515 | Unique product for human FLOT1 (RefSeq: NM_001318875.2) | Hryniewicz-Jankowska, Anita et al. Data Brief. 2025. |
| KIBRA | CTCCGAGGCCAGAGCTGTAAGGAAC | CTAGAGGACTTGTGACTCAGTAC | 223 | Unique product for human WWC1 (RefSeq: NM_015238.3) | Yuan X, et al. J Extracell Vesicles. 2022. |
| MVP | ACCTGCACTTCTAGATTCATCTTCT | CCTGATGATGGTGGCCTGAAT | 558 | Unique product for human MPV (RefSeq: NM_005115.5) | Li, Chunlin et al. Aging. 2024. |
| Syndecan1 | TCGAATCTCTGTGCCTTCGTC | AAACCTTGGCTGAACCTACCG | 122 | Unique product for human SDC1 (RefSeq: NM_002997.5) | Yuan X, et al. J Extracell Vesicles. 2022. |
| p53 | CAGCACATGACGGAGGTTGT | TCATCCAAATACTCCACACGC | 125 | Unique product for human TP53 (RefSeq: NM_001407269.1) | Xiao X, et al. Signal Transduct Target Ther. 2021. |
| p16 | GATCCAGGTGGGTAGAAGGTC | CCCCTGCAAACTTCGTCCT | 74 | Unique product for human CDKN2A (RefSeq: NM_058197.5) | Xiao X, et al. Signal Transduct Target Ther. 2021. |
| p21 | TGTCCGTCAGAACCCATGC | AAAGTCGAAGTTCCATCGCTC | 139 | Unique product for human CDKN1A (RefSeq: NM_000389.5) | Xiao X, et al. Signal Transduct Target Ther. 2021. |
| GAPDH | GAACGGGAAGCTCACTGG | GCCTGCTTCACCACCTTCT | 123 | Unique product for human GAPDH (RefSeq: NM_001289746.2) | Xiao X, et al. Signal Transduct Target Ther. 2021. |

Supplementary Table S1. PCR primer sequences for mRNA
